# A New Paradigm in AC Drive Control: Data-Driven Control by Learning Through the High-Efficiency Data Set—Generalizations and Applications to a PMSM Drive Control System

**DOI:** 10.3390/s24227313

**Published:** 2024-11-15

**Authors:** Madalin Costin, Ion Bivol

**Affiliations:** 1 Department of Electrical Engineering and Energy Conversion Systems, “Dunarea de Jos” University of Galati, 800008 Galati, Romania; ion.bivol@ugal.ro; 2 Department of Automatic Control and Applied Informatics, “Gheorghe Asachi” Technical University of Iasi, 700050 Iasi, Romania

**Keywords:** data-driven control (DDC), model predictive control (MPC), permanent magnet synchronous machine (PMSM), searching algorithm, radial basis function (RBF) neural networks (NN), interpolation technique

## Abstract

This paper presents a new means to control the processes involving energy conversion. Electric machines fed by electronic converters provide a useful power defined by the inner product of two generalized energetic variables: effort and flow. The novelty in this paper is controlling the desired energetic variables by a Data-Driven Control (DDC) law, which comprises the effort and flow and the corresponding process control. The same desired useful power might be obtained with different controls at different efficiencies. Solving the regularization problem is based on building a knowledge database that contains the maximum efficiency points. Knowing a reasonable number of optimal efficiency operation points, an interpolation Radial Base Function (RBF) control was built. The RBF algorithm can be found by training and testing the optimal controls for any admissible operation points of the process. The control scheme developed for Permanent Magnet Synchronous Motor (PMSM) has an inner DDC loop that performs converter control based on measured speed and demanded torque by the outer loop, which handles the speed. A comparison of the DDC with the Model Predictive Control (MPC) of the PMSM highlights the advantages of the new control method: the method is free from the process nature and guarantees higher efficiency.

## 1. Introduction

Electric Drive Systems (EDSs) have recently come into focus in regard to developing advanced technologies in digital processors, signal processing, and power systems. The main benefits provided by developing EDSs can be summarized as follows: fast transients, high power density, large range of speed and power, low cost, safe operation, robustness, modular components, etc.

The increasing number of EDS applications (see automotive) implies new control techniques aimed at improving the performance of the energy conversion process. Firstly, the efficiency improvement of the EDSs can be done from the conception phase by using the Permanent Magnet Synchronous Machine (PMSM), which has high efficiency as a consequence of the use of modern technologies for manufacturing permanent magnets. Secondly, the EDS efficiency increase with PMSM may be obtained by using an adequate optimal control law designed in accordance with a certain methodology.

The traditional control strategies are based on the physical equations of the process model; for example, the ones commonly used in EDSs are Field Oriented Control (FOC) with the mathematical model in an appropriate coordination frame that is used by adequate decoupled control changes for torque and flux, the loops being usually designed with classical PI linear controllers [1,2], Direct Torque Control (DTC)—a bang to bang control implemented by hysteresis controllers [2], and Model Predictive Control (MPC), which uses a future control sequence obtained by the process model predictor aimed at finding the best control law sequence by minimizing a known objective function [3].

The specific particularity of the advanced EDSs is that the input voltage and frequency are not directly applied from the power grid, which exhibits fixed values. The command is obtained using a power interface consisting of an electronic power inverter, which allows the generation of variable values of the voltage and frequency to obtain the required performance [4]. This task is crucial for getting advanced results, which can improve the closed-loop performance of the EDSs [5,6].

In the past few years, the MPC control strategy has been extensively developed and improved, being an actual motivation for research and industry [7,8,9,10,11]. More advanced studies are dedicated to improving control results, taking into account the nonlinear magnetic circuit [12], advanced optimization method [13], robustness [14], or fault-tolerant capability [15], respectively. However, the MPC strategy is strongly based on the process model, which in practice can differ from the one of the real systems, resulting in lower control performance.

In EDSs, Artificial Intelligence Methods (AIMs) are well approached in control systems. Thus, torque minimization was successfully obtained by using the Neural Network (NN) method [16], the Radial Basis Function Proportional-Integral-Derivative (RBF-PID) controller developed in [17] led to improved tracking results, and an iterative learning control strategy reduces the torque ripple [18]. More advanced AIMs can be found in the survey [19].

Reinforcement Learning (RL) is a popular advanced framework used to control systems and devices in a wide range of applications because of its ability to autonomously find control policies able to achieve a desired goal without assuming that the environment is known. Thus, in [20], it is proved that the actual methods of deep RL led to obtaining a suitable control structure for the adaptive controller. Further, a low-complexity gradient descent solution with a backtracking iteration approach for the MPC Quadratic Programming (QP) problem leads by minimizing to reducing the number of the searched control inputs and improving the control performances [21]. In spite of the various benefits obtained by AIMs, these control algorithms require a large amount of hardware and software resources.

A new trend in the control theory applications is based on Data-Driven Control (DDC), which discusses the transition from Model-Based Control (MBC) applications to Model-Free Control (MFC) adaptive structures involving learning in a given control framework context [22,23,24,25]. The DDC method is an advanced strategy that can learn the dynamics [26] or can allow for obtaining different formulations via regularizations and relaxation methods, bridging the direct and indirect data-driving control [27]. Indirect data-driven control is formulated as a bi-level optimization problem: first, a model is fitted from the data in the inner identification before the model is used to control the outer problem. The model is used for many reasons, such as the availability of thorough analysis and design of the control methods. Another advantage is that the models are very compressed compared to the data-driven representation, and last but not least, they provide more details about the stabilization, optimality, and robustness behavior of DDC [28].

Also, mention may be made that predictive control applications can be obtained directly resulting from the data set, which does not include all the main features of the classical predictive control strategy, such as the integrator that ensures an offset-free control, constraint handling, feedforward action, and disturbance control model, which make the strategy more versatile for real-time applications [29]. Furthermore, an algorithm designed to control an unknown stochastic linear time-invariant system illustrates the high closed-loop performance obtained by a data-enabled predictive control strategy with a novel, robust distribution [30]. A simultaneous MPC and DDC strategy with relaxed assumptions on the model in a proper framework shows how a nonconvex optimal problem is transformed into one with convex constraints [31]. The input-mapping-based data-driven model predictive control is successfully used for systems with bounded disturbance [32] or for advanced learning [33]. For generalization, ref. [34] discusses a data-enabled predictive control parametric algorithm that combined the identification method obtained by a learning procedure and control of one of the dynamical systems, resulting in an equivalent MPC formulation that is valid for both linear time-invariant systems, which are directly applied, and nonlinear systems that require a regularization procedure.

As expected, an emerging technology in control system development may be based on both DDC and MPC strategies. Although EDSs are a rapid process for control applications, with the help of advanced sensors and adequate numerical signal processing, a large amount of data can be collected and processed, which is sufficient for implementing the DDC solution. Thus, based on White-Box (WB) modeling estimation by the least squares technique, in [35], a DDC-MPC method is proposed for the current controlling of PMSM, which leads to compensating the modeling errors and improving the control performance for both dynamic and steady-state regimes. A relatively recent paper [36,37] deals with the transition from the classical model-based approach towards data-driven optimal control strategies, which take into account the model predictive control paradigm, leading to the data-enabled predictive control. Finally, ref. [38] shows that a fractional PI controller for a PMSM drive can be obtained by a DDC algorithm in certain practical conditions.

The MPC strategy and the more recently inaugurated DDC have solved many problems. An important issue in the EDSs with PMSM is related to the efficiency improvement by control means. The literature [39,40,41] contains a variety of solutions for improving the efficiency of EDSs with PMSM. Most of them are based on the modeling of the power losses with algebraic relations, taking into account some specific constants of materials. The minimization of an analytic cost function with constraints will give the optimal solution that ensures high efficiency. This approach has an elegant mathematical description but also many limitations: the constants of the materials depend on the magnetic/electric fields, there are losses that cannot be modeled analytically (abnormal losses), and many methods are designed for partial losses.

Finally, extensive literature is focused on the MFC strategy (philosophy). Research started early in 1956 through the project [42] “A proposal for the Dartmouth Summer Research Project on Artificial Intelligence” by J. McCarthy, Dartmouth College, M. L. Minsky, Harvard University, N. Rochester, IBM Corporation, C. E. Shannon, Bell Telephone Corporation. It is concluded in this research project: “If a machine can do a job, then an automatic calculator can be programmed to simulate the machine’’. By using a ”mutatis mutandis” paraphrase, we may say: “If a classic automatic control based on a physical model can control a process, then a computing system can control the same process without a physical model.” This is the main principle in this paper, aiming to show how an MBC strategy offers relatively good possibilities to develop an MFC in certain energetic constraints. The present paper makes a transition from the MBC to MFC in the DDC paradigm for high-performance objectives.

For a large set of physical processes, the main controlled quantities are effort and flow. Their product is the useful power. In the paper, we develop a new technique to find adequate controls of the effort and flow in terms of maximum efficiency, that is, minimum losses. These appropriate controls are extracted from an apriori DataBase (DB) in terms of required effort and measured speed. The apriori DB mainly contains the steady-state data in the open loop tests for the speed, the effort, and the corresponding controls. An RBF interpolative system is trained so that, based on a few sets of optimal data, it can extract the controls for any desired speed and flow. In the online operation of the process, the initial DB is adaptive. An online optimization system searches for new optimal controls when the process parameters are changed. DB is free from process parameters and, hence, from the process model.

The paper shows that a computerized system based on an experimental database, without knowing the parameters of the process, can do the same job as an MPC control system with multiple advantages. Thus, we propose a new combined linear PI and DDC, where the control variables are directly obtained from an experimental database by an RBF methodology. The key to DDC is to find the relation of the energetic variables with the control variables by an RBF-NN interpolative surface. These surfaces are built with a set of optimal support points extracted from a large knowledge database.

In the following, we demonstrate that the proposed combined linear PI and the RBF-NN methodology can successfully perform a dynamic simulation of EDSs with PMSM, controlled by the MPC methodology, without knowing any parameters of the motor or driven process.

In accordance with the state of the art, the present paper brings the following new contributions:

(i) We propose a new combined linear PI and DDC control methodology that uses the process control variables obtained from a database of design, experimental, and simulated data built by an RBF technique.
(ii) In the case of the EDSs, the combined control methodology deals with an outer time domain PI control of speed and an inner implicit RBF control loop.
(iii) The inner control loop is based on a database, which contains the optimal controls corresponding to any speed and torque of process operation points. The control law is in implicit form, which is a set of high-efficiency steady-state process operation points.
(iv) The RBF methodology compresses the set of all admissible operation points by network architecture, with a few optimal data basis functions.
(v) We have evaluated the performance of the new DDC algorithm by simulating the PMSM control scheme, in which the demanded torque by the PI controller and the measured speed are the inputs for the RBF inner loop. The output of the inner loop is the inverter’s command shaping input, which selects the proper voltage vector sequence of the invertor.
(vi) The inner RBF control loop is adaptive, that is, the inverter control is temperature and magnetic saturation dependent because the parameters of the process change with exogenous conditions.

The organization of this paper is structured as follows. Section 2 shows how to obtain a data-free control algorithm based on the physical modeling of the processes. Section 3 is dedicated to the DDC of AC drives, where the illustrative application of PMSM drive control is made. The comparative results obtained by MPC and DDC techniques for a PMSM are seen in Section 4. Finally, Section 5 summarizes the main benefits obtained by the newly proposed control method.

## 2. Problem Statement—From Classical Physical Modeling of Energy Conversion Flow to Data-Free Model Control

### 2.1. Problem Statement

**Figure 1:**
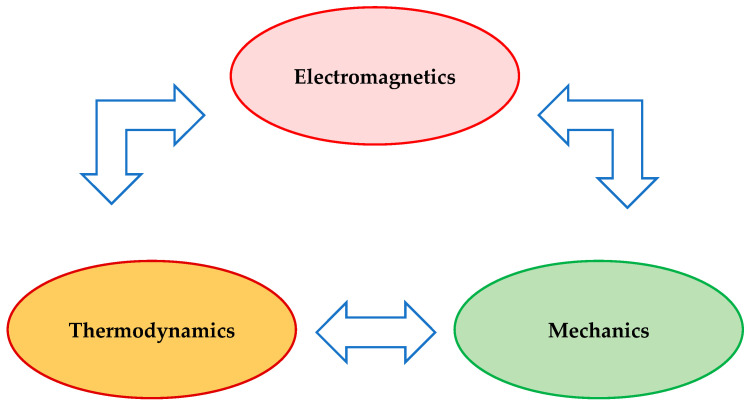
The diagram of the macroscopic energy flow conversion.

The energy conversion flow is an essential objective for ensuring social and economic stability. In industry and residence, many applications are designed to deal with the macroscopic energy conversion that implies three main types of fields: electromagnetics, mechanics, and thermodynamics (Figure 1).

Starting from these energy conversion possibilities, their different conversion processes can be developed in practice, depending on adequate constraints.

In the various physical processes (electric, mechanical, hydro- or thermal ones), the useful work desired is universally characterized by a pair of energetic variables: the effort *e*(*t*) and the flow *s*(*t*). The inner product of these generalized variables is the instantaneous useful power:

(1) $$p_{2}(t)=e(t)\cdot s(t).$$

The different forms of primary power *p*_1_(*t*) can be converted into useful forms by various means. Let *η* be the efficiency of the power conversion process. Then, the primary power *p*_1_(*t*) is easily obtained:

(2) $$p_{1}(t)=p_{2}(t)/\eta .$$

If, for example, we refer to a mechanical process of motion that develops a useful force *f_u_* and a linear velocity *v*, the motion being described in external (Cartesian) coordinates, then we have *e* = *f_u_*, and *s* = *v*. If the motion is rated to the drive motor shaft, then *e* = *m* and *s* = *ω*, where *m* is the developed torque and *ω* the angular speed. In the case of an electrical process, the electrical voltage represented in the complex domain is $e=\bar{u}$ and the corresponding current is $s=\bar{i}$. Similarly, in the case of Hydro, Ventilation, and Air Conditioning (HVAC) processes, we get the pressure *e* = *P* and the flow rate *s* = *Q*.

The energetic macroscopic process described above is usually well-modeled by the actual intrinsic physical theories. In the control theory, the use of this model with lumped parameters has some vulnerabilities: high sensitivity to parameter variations, incomplete modeling of some loss components, linearity on a small range, low control accuracy at high frequencies, etc. However, analytic modeling has the advantage of simplicity, being, in many approaches, a starting point in developing advanced control methods.

**Figure 2:**
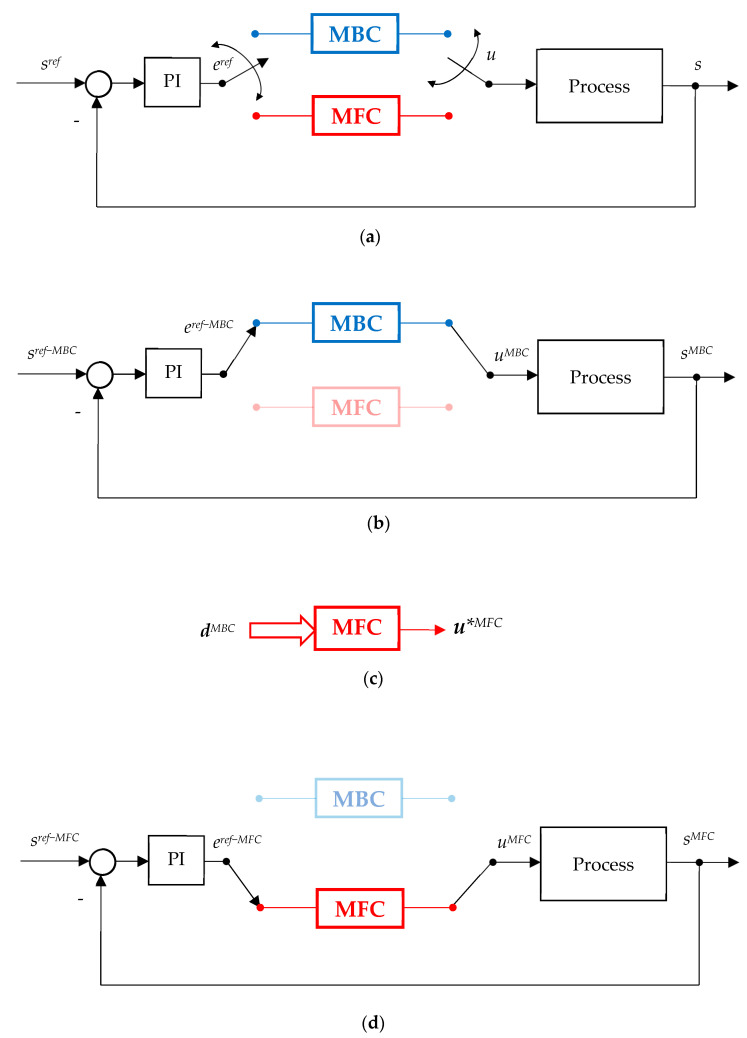
The transition from MBC to MFC: (**a**) The basis control scheme; (**b**) Stored data from MPC running; (**c**) Designing of MFC strategy: trainning phase; (**d**) Designing of MFC strategy: testing phase.

The transition from MBC to model MFC is shown in Figure 2. To this purpose, a combined PI and inner control (cascaded or not) are considered for a given process described by adequate physical model equations.

The inner control is switched from MBC to MFC whenever necessary, giving the control *u* for the output, as seen in Figure 2a.

In the first stage, shown in Figure 2b, the inner control is switched to MBC. At this step, a collection of the main data in a steady-state regime was selected, corresponding to high efficiency, grouped into:

(3) $$d^{MBC}={\left[\begin{array}{cccc} u^{MBC} & y^{MBC} & e^{MBC} & s^{MBC} \end{array}\right]}^{T},$$

where it was considered that in steady-state regime *e^ref^ = e.*

The next step is the design of the MFC controller. This controller has an interpolative-adaptive structure with a variable structure, being able to learn from MBC data and having as input the data stored (3) in a sufficient number, and the output u*^MFC^ as shown in Figure 2c. The MFC algorithm is trained in an open structure and has the ability to learn from the experience given by MBC.

Having closed the training phase of MFC, the testing phase is performed in the closed-loop structure as indicated in Figure 2d.

The transition from MBC to MFC depends on the process that is subject to the control strategy. This procedure may be applied to the process with complex, nonlinear, and multivariable structures.

### 2.2. The DDC Strategy of the Energy Conversion Process

The MFC technique is a modern approach with large applicability in practice. From MFC control techniques, a particular direction is described by the DDC strategy, to be explored below.

The numerical processing of the signals that refer to the training phase is carried out by the MBC strategy, which is why the MBC acronym will be ignored in the symbolization of signals, distinguishing between the acronyms DDC and MPC whenever necessary.

The state model of this nonlinear deterministic conversion process is described by:

(4) $$\left\{\begin{array}{l} \dot{x}=f(x(t),u(t),\theta (t))\\ y=g(x,u) \end{array}\right..$$

The state **x**, control input **u**, and output **y**, respectively, are restricted to the sets of adequate dimensions: $x(t)\in X\subset {\mathbb{R}}^{N}$, $u(t)\in U\subset {\mathbb{R}}^{M}$ and $y(t)\in Y\subset {\mathbb{R}}^{P}$. Further, the parameters of the process are compressed within the vector ***θ***(*t*). The nonlinearity of the state model (3) is emphasized by the nonlinear functions **f** and **g**.

Let us adopt the following output variables:

(5) $$\begin{array}{l} y={\left[\begin{array}{cccc} e & s & p_{1} & p_{2} \end{array}\right]}^{T}\\ h(x,u)=p_{1} \end{array}.$$

By common discretizing techniques, the corresponding discrete state space model of the plant (4) becomes:

(6) $$\left\{\begin{array}{l} x(k)=f(x(k-1),u(k-1,\theta ,T_{s})+\xi \\ y(k)=g(x(k),u(k)) \end{array}\right.,$$

where the discrete states **x**(k), control inputs **u**(k), and outputs **y**(k) are matrices of appropriate sizes.

Also, in (6), *ξ* is the discrete noise of the process, while *T_s_* is the sampling time period.

The control problem to be solved is defined as follows: seek a control **u**(t) that will yield the useful work desired:

(7) $$E_{2}(t)=\int_{t_{0}}^{t_{1}}p_{2}(t)dt,$$

when the input of the system is supplied by the energy:

(8) $$E_{1}(t)=\int_{t_{0}}^{t_{1}}p_{1}(t)dt,$$

with a natural condition resulted from (2):

(9) $$\left.\begin{array}{r} E_{1}(t)=E_{2}(t)/\eta \\ \eta <1 \end{array}\right|\Rightarrow E_{2}(t)<E_{1}(t),$$

such as for any other control $\hat{u}(t)$, which also brings the same work *E*_2_, the energy *E*_1_ is higher:

(10) $$\int_{t_{0}}^{t_{1}}h(\hat{u},x)dt<\int_{t_{0}}^{t_{1}}h(u,x)dt.$$

In other words, we assume that for a given desired pair of the generalized variables *e*(*t*) and *s*(*t*), there exists a bounded control **u** and a bounded cost criterion, which can be expressed either in a continuous set formulation:

(11) $$C_{c}=\left\{\left.\int_{t_{0}}^{t_{1}}\varepsilon^{2}dt;\int_{t_{0}}^{t_{1}}h(u,x)dt;THD_{e}\right|E_{2}(t)=cst.\right\},$$

or in a discrete representation:

(12) $$C_{d}=\left\{\left.\sum_{k=1}^{N}\varepsilon^{2}(k)T_{s};\sum_{k=1}^{N}h(k)T_{s};THD_{e}\right|E_{2}(k)=cst.\right\},$$

where the mean square error was used: $\varepsilon^{2}={\left(e^{ref}-e\right)}^{2}+{\left(s^{ref}-s\right)}^{2}$.

Also, in the cost formulation (11) and (12), the Total Harmonic Distortion (THD) factor is used, which is a measure of harmonic distortion computed by:

(13) $$THD_{e}=\frac{\sqrt{\sum_{n=2}^{\infty }E_{n,RMS}^{2}}}{E_{f,RMS}},$$

where $E_{f,RMS}$ and $E_{n,RMS}$ stand for the Root Mean Square (RMS) values of fundamental and harmonic components of the effort *e*, respectively.

For the same desired work (7), the final cost ***C***, which is lower, has better control *u*. The interval (*t*_1_ − *t*_0_) length is a time window with the required energy *E*_2_ (7), where *t*_1/0_ is the end/start time.

It can be mentioned that the input power may be either positive $p_{1}>0$ or negative $p_{1}<0$, as the power is delivered or received through the process.

The first term in (11) and (12) refers to the control quality in the control horizon *N* length, and the second term, respectively, refers to the input energy.

Numerical computation has large applicability in data computation. For our approach, we define the cost-solving problem based on (12) as follows:

(14) $$\begin{array}{l} C_{\min}=\underset{u,x}{\arg\min}\;C_{d}\\ s.t.:\;\left\{\begin{array}{l} E_{2}=cst.\\ \eta (k)=\max \end{array}\right. \end{array},$$

where the constraints *E*_2_ and *η* ensure the uniqueness of the optimal cost problem.

The process model (4) is in the time domain, having an explicit form. A more convenient approach is developed in the following.

We introduce the implicit reduced model:

(15) $$\left\{\begin{array}{l} G(\overset{⏝}{y},u)=0\\ \overset{⏝}{y}={\left[\begin{array}{cc} e & s \end{array}\right]}^{T} \end{array}\right..$$

The control law **u** results by inversion of the model (15)

(16) $$\left\{\;\begin{array}{l} u=F(e,s)\\ F=G^{-1} \end{array}\right.,$$

The processes analyzed in the paper have nonlinear devices with finite states as inverters. We adopted an algorithmic function **F**, which operates with the specific DDC method based on DB.

In model (15), the $\overset{⏝}{y}$ has a reduced dimension $\dim(\overset{⏝}{y})<\dim(y)$. In consequence, the output variables *p*_1_ and *p*_2_ are free, being unconstrained, and the resulting control **u** is not unique.

A regularization procedure is done by selecting from the experimental data a finite set {e,s,**u**} corresponding to the operation points at maximum energy efficiency.

Let us consider a time window $\left[t_{0},t_{1}\right]$ for the requested output trajectories of both generalized variables:

(17) $$\left\{\begin{array}{l} g_{1}(t)=e(t)\\ g_{2}(t)=s(t) \end{array}\right.,\;t\in [t_{0},t_{1}].$$

By eliminating the time from relations (16), we have the implicit representation:

(18) $$f_{\varepsilon }(e,s)=0.$$

Each discrete point *kT_s_*, with *k* = 1, …, *N_obs_*, where *N_obs_* is the number of observation points, on the trajectory (17) in the generalized variables (*e*, *s*) plane, has the input controls, which represent the support points for the control surfaces:

(19) $$\begin{array}{l} u(k)=f(e(k),s(k))\\ u=[\begin{array}{cc} u_{1} & u_{2} \end{array}]\;\\ f=[\begin{array}{cc} f_{1} & f_{2} \end{array}] \end{array}.$$

The surfaces (19) in the continuous domain represent the endless set of steady-state operation points. Any stable operating point (*e*, *s*) is correlated with the corresponding controls (*u*_1_, *u*_2_). A trajectory of motion (16) within the permissible range, beginning in a stable initial state (*e*_0_, *s*_0_) and reaching the final state (*e_f_*, *s_f_*), passes through a countless set of intermediate energy states since in classical physics, it is admitted that states of physical processes can vary only continuously. By using digital process control techniques, the set of states traveled from the initial state to the final state becomes countable due to sampling operations. An exhaustive stability condition of the process controlled by the two fundamental energy quantities, effort *e*, and flow *s*, is as follows: if the speed control loop is stable in the sense that the required effort *e*^ref^ is on the control surfaces (19), the operating points on the motion path will be stable. Through the sampling process, the better the stability, the longer the sampling period.

Generalizing, we can say that the multivariable control law (19) can be generated with the required precision using a known set $M_{F}$ of operating points of the process:

(20) $$M_{F}={\left[\begin{array}{cc} \overset{⏝}{y} & u \end{array}\right]}^{T}.$$

Through an interpolation algorithm $A_{F}$ associated with the $M_{F}$ database, for any current value $\overset{⏝}{y}\in Y$, a value u∈U is generated that approximates the control law $F(\overset{⏝}{y})$ relation (15) so that:

(21) $$u=A_{F}(\overset{⏝}{y}).$$

The control law is determined by means of an experimental data set $M_{F}$ and some continuity and smoothing properties of the surfaces (15), which are the specific properties of energetic inertial processes.

In summary, the control law has the form:

(22) $$\begin{array}{l} u=A_{F}(\overset{⏝}{y})\\ u,\overset{⏝}{y}\in M_{F} \end{array},$$

with properties:

(23) $$\begin{array}{l} u(k)=A_{F}(\overset{⏝}{y}(k)),\;\forall (u(k),\overset{⏝}{y}(k))\in M_{F}\\ \;k=1,2,\ldots ,N_{obs} \end{array}.$$

**Figure 3:**
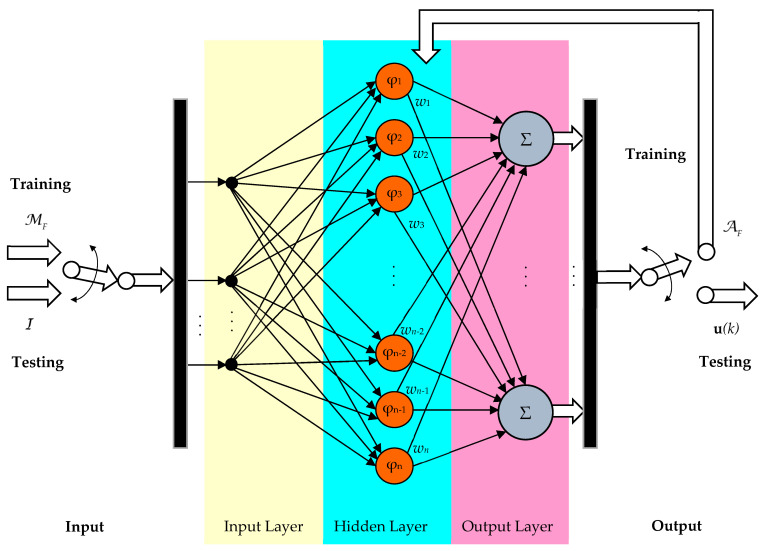
The RBF-NN used for obtaining the plant input.

The RBF-NN is largely used in control systems [17,43,44]. A typical RBF-NN structure used for obtaining the input plant command is shown in Figure 3.

The RBF-NN is used for two objectives, training and testing:

(24) $$\begin{array}{l} \mathrm{Training}:\;A_{F}=RBF(M_{F})\\ \mathrm{Testing}:\;u^{RBF}(k)=RBF(A_{F},(e(k),s(k)))\\ \;\;\;\;\underset{\;\mathrm{Input}\;}{↔}\;\underset{\;\mathrm{Output}\;}{↔} \end{array}.$$

where RBF means the function of the NN.

Having a classical structure, the RBF is organized into three levels of layers: input, hidden, and output layers, respectively. Moreover, the weights obtained in the testing step are grouped into:

(25) $$A_{F}={\left[\begin{array}{ccccc} w_{1} & w_{2} & \ldots & w_{n-1} & w_{n} \end{array}\right]}^{T}.$$

The output of the RBF-NN is given by a linear combination:

(26) $$u^{RBF}(k)=A_{F}^{T}\varphi =w_{1}\varphi_{1}+w_{2}\varphi_{2}+\ldots +w_{n}\varphi_{n},$$

where the activation function is given by Gaussian distribution

(27) $$\varphi_{l}=e^{-\frac{{\left\|I_{l}-\mu_{l}\right\|}^{2}}{\sigma_{l}^{2}}},\;l=1,n.$$

having the parameters σ and μ, which are the basis width vector and center points of the Gaussian function, respectively.

Also, the input Gaussian function is represented by the set:

(28) $$I=\{A_{F},e^{ref}(k),s(k)\}.$$

**Figure 4:**
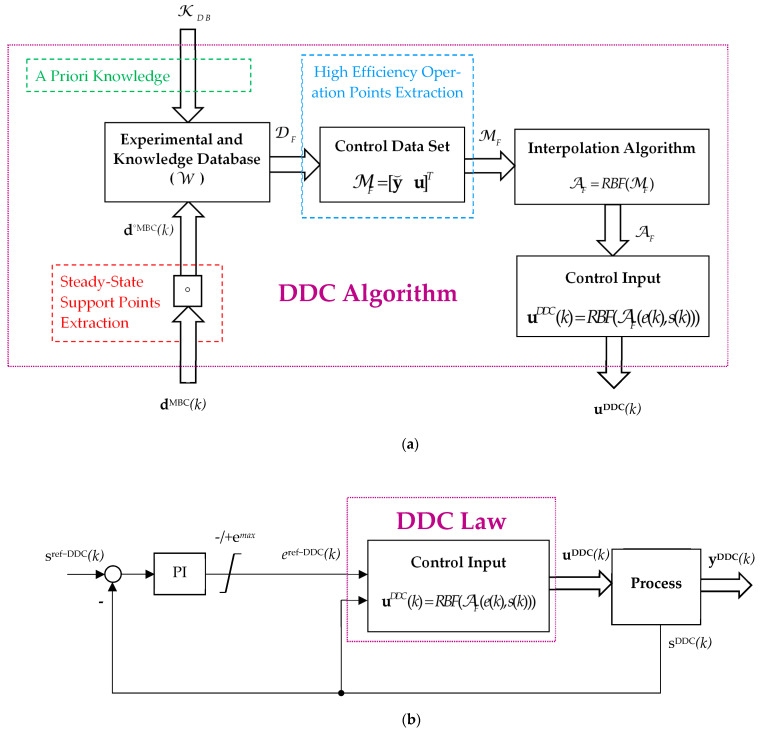
A combined control structure with linear PI outer control loop and DDC inner control. Designing of DDC strategy: (**a**) Training phase; (**b**) Testing phase.

Enhancing the RBF depicted in Figure 3, Figure 4 illustrates the combined control structure of a generic process that involves an outer explicit flow control loop and an inner multivariable data-driven control loop.

The fundamental operation key of the DDC schema is the knowledge database. This database contains a large set of process operation points, among which there are limited steady-state ones. In the block scheme in Figure 3, the actual values $d^{MBC}(k)$ of control, output, effort, and flow are compressed in the set:

(29) $$d^{MBC}(k)={\left[\begin{array}{cccc} u(k) & y(k) & e(k) & s(k) \end{array}\right]}^{T},$$

where **y**(k) is the output of the process, and *N* is the number of collected data.

The next task is to select a sufficient number of steady-state points $d^{{}^{\circ}MBC}(k)$ from all the operation points from $d^{MBC}(k)$,

(30) $$\begin{array}{l} d^{{}^{\circ}MBC}(k)={\left[\begin{array}{cccc} u^{{}^{\circ}}(k) & y^{{}^{\circ}}(k) & e^{{}^{\circ}}(k) & s^{{}^{\circ}}(k) \end{array}\right]}^{T},\;\\ \mathrm{with}:\;\left\{\begin{array}{l} d^{{}^{\circ}MBC}(k)\subset d^{MBC}(k)\;\\ \frac{\partial d^{{}^{\circ}MBC}(k)}{\partial t}\equiv 0(k)\;\\ 0(k)={\left[\begin{array}{cccc} 0(k) & 0(k) & 0(k) & 0(k) \end{array}\right]}^{T} \end{array}\right., \end{array}.$$

To obtain a sufficient number of steady-state points contained in the set $d^{{}^{\circ}MBC}(k)$, the operator used for data extraction will be symbolized by “°”.

In addition, the apriori information included in the set is considered as input data:

(31) $$K_{db}=\{R_{N};E_{N};H_{N}\},$$

where: the rated specifications of the process are contained in the set $R_{N}$, the environment and design requirements, which are contained in the sets $E_{N}$ and $H_{N}$, respectively.

The input control data set will be computed based on both aprioric information and process data. In fact, the design of the required database is quite complex and will be presented in the next section for the case of a practical application. However, starting from the inputs of knowledge $K_{DB}$ and data $d^{{}^{\circ}}(k)$, the output of the database is:

(32) $$D_{F}=W\{K_{db};d^{{}^{\circ}MBC}(k)\},$$

whereby W stands for a numerical operator used for data processing in the database.

The proposed PI and combined DCC structure are seen in Figure 4.

The training phase design of the DDC strategy is shown in Figure 4a, where the main steps of the DDC algorithm are highlighted, while the testing phase performed in a closed loop structure is shown in Figure 4b.

The DDC control structure in Figure 4b is designed for tracking the output of the process s^DDC^(k) to its reference s^ref-DDC^(k) by using a linear PI controller, having the output e^ref-DDC^(k) bound within the limits −/+e^max^.

The control data set $M_{F}$ contains the maximum efficiency operation points selected from the knowledge DB. The interpolation algorithm A_F_ finds the actual control ***u***(k) based on the required effort *e*(*k*)*^ref^* and the measured flow *s*(k).

We assume that all points $M_{F}$ (19), also referred to as nodes or “support points”, are distinct and not collinear. Due to the intrinsic nature of the energy conversion process, the DDC law (16) is geometrically represented as continuous differentiable surfaces, which allows us to find an interpolation algorithm $A_{F}$, so that these surfaces pass exactly through each experimental point from $M_{F}$ (18). The maximum efficiency operation points (*e*, *s*) are selected from the knowledge DB by a grid (direct) search method. These methodologies involve setting up grids in the input space (*e*, *s*) and evaluating the power efficiency of each grid point. The operation point that corresponds to the maximum efficiency was considered the best solution.

Two interpolation methods that are widely used in different application fields (in areas such as computer graphics, physical modeling, geographic information systems, medical imaging, and more) have been adopted. We used two basic methods: Delaunay triangulation and related methods and RBF-NN interpolation.

Given a set of data, we want to find a rule that allows us to deduce information about the process we are studying at locations that differ from those at which we performed our measurements.

The interpolated value of a point, other than the support points, was obtained by local interpolation techniques with the three nearby points. One commonly used approach is the Delaunay triangulation of data and the Voronoi diagram of a set of points, which is the dual of the first approach (Delaunay triangulation).

The RBF network has its origin in performing the interpolation of a set of data points in a multidimensional compact domain with arbitrary accuracy, given a sufficient number of data points. It has a network architecture with weighted basis functions. The solution is optimal by minimizing functional containing regularization terms. The RBF method compresses a very large volume of data with the help of a much smaller number of weighted basic functions in the training process. In the exam (assessing) stage, the RBF network returns any point in the field covered by the data used in the training stage by an interpolation process of the weighted basis function. The discrete model of the control law is practically non-inertial because the control *u*(*k*) at the current step *k* is obtained by accessing the fast interpolation algorithm $A_{F}$:

(33) $$\begin{array}{l} u(k)=A_{F}(y(k)),\\ \;k=1,2,\ldots ,N,\\ N=T_{d}/T_{s}, \end{array}$$

where *T_d_* is the desired final time of the motion trajectories.

The new control methodology of the energy conversion process in terms of the effort and flow is shown in Figure 4.

The goals (11) and (12) are attained by two control loops: a MIMO inner control loop for the desired effort *e^ref^*(*t*) and measured flow *s*(*t*) and an outer classical PI speed control loop.

## 3. The DDC of AC Drive—Illustrative Case Study: The DDC of a PMSM Drive System

The control method proposed in the previous section can offer a new alternative to the classical methods, which are based on model accuracy. The novel approach is model-free, being suitable for a large class of applications.

In the following, we start by applying the principle of the DDC scheme to AC drives. The next step is an illustrative application for a PMSM driving system controlled according to the methodology developed in this paper. As the MBC method, the MPC strategy was chosen due to its ability to provide an optimal solution without any frequency modulator.

### 3.1. The DDC Principle of AC Drives

AC drives are extensively found in applications due to their high performances in dynamic regime.

Usually, the cascade control structure of AC drives is used to separate the different dynamics of electrical and mechanical parts, respectively, and then to have an independent control of each loop.

In addition to the advanced control law, most attention is paid to the electronic power inverter used to obtain the voltage supply. This aspect will be largely approached in the following sections.

This section is dedicated to the DDC principle applied to AC drives.

As mentioned in the second section, the DDC method is based on a learning technique. For our approach, the learning is based on the collected data from running the MPC strategy.

**Figure 5:**
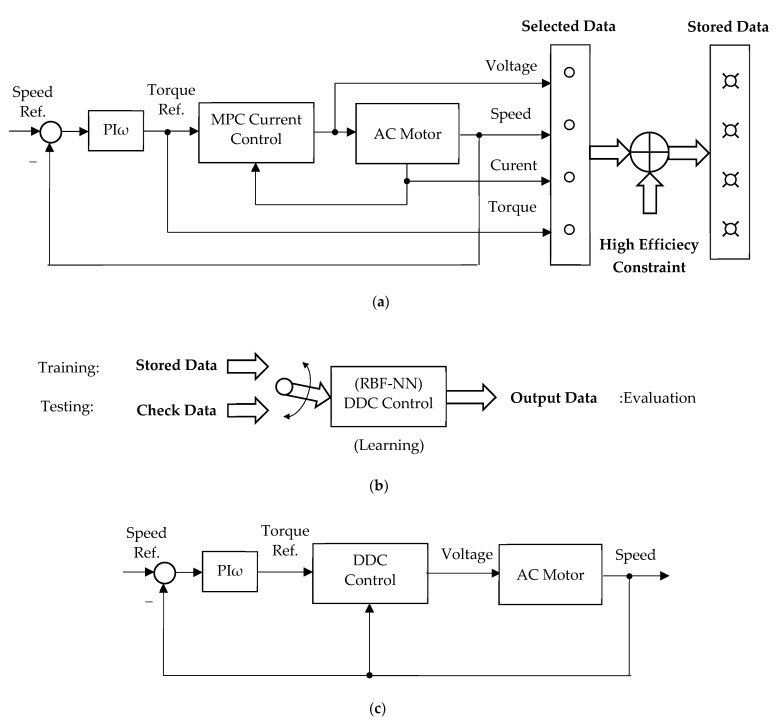
The principle of the DDC strategy applied to an AC motor: (**a**) Stage I: Stored data from MPC cascade control running; (**b**) Stage II: Training of testing the RBF-NN DDC control; (**c**) Stage III: Testing of the DDC control.

The stages of DDC design are seen in Figure 5.

It starts from a cascade structure, having the well-known quantities: speed (actual and reference), torque (reference), voltage, and current (components). A brief description of each stage can be done as follows:

- Stage I: Stored data from MPC cascade control running. In this stage, we selected a limited data set obtained from the MPC cascade running in a steady-state regime for speed, torque, voltage, and current. Then, we impose the constraint of high efficiency, resulting in the stored data;
- Stage II: Training of DDC strategy. The stored data obtained in the previous stage serves as a base in this training stage for DDC design, which is done in an open structure. Usually, the stored data are numerically processed in order to improve the main features: efficiency, robustness, tracking, and disturbance rejection. These objectives are achieved by using a DB, which offers multiple possibilities for data processing;
- Stage III: Testing of the DDC method. Learning from the previous stage, the DDC strategy is able to operate at any point required by the application of AC drive. The DDC method is implemented with no cascade control structure, as shown in the figure.

The DDC strategy may be a viable solution in the actual AC drives whose model parameters sensitivity is still reported as an open problem.

Nevertheless, an important objective is the improvement of efficiency in various scenarios.

Usually, the electrical parameters of the model of AC drives are subject to variations as dependent on exogenous conditions that can differ with environmental conditions: temperature, external fields, altitude, etc. In this case, the robustness of the DDC strategy must be studied under these circumstances.

### 3.2. DDC of the PMSM Drive System

**Figure 6:**
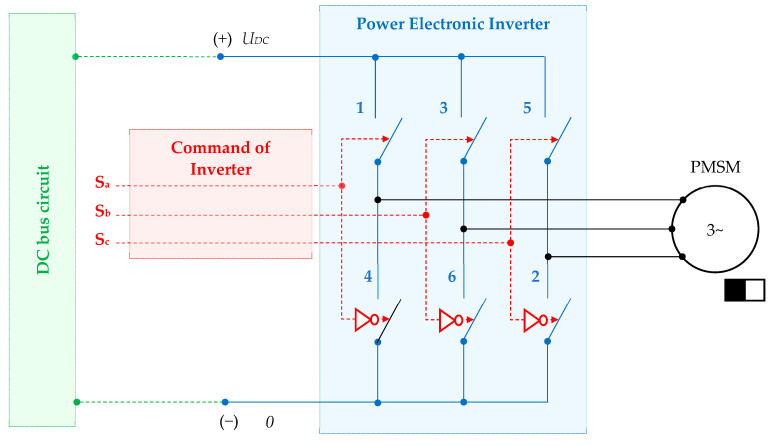
Power structure of the PMSM drive.

The PMSM drives are largely used in the adjustable applications that require high-performance control performances and high efficiency. A conventional structure used in practice includes a two-level power electronic inverter, as shown in Figure 6. This input of the power electronic inverter usually ensures a constant DC bus voltage *U_DC_*, which is provided by a DC bus circuit that contains a DC link voltage and a voltage rectifier. The main feature of this power structure is that the discrete logic command **S**_abc_ = {S_a_,S_b_,S_c_} applied on the top switches {1,3,5} is negated when applied to the bottom switches {4,6,2}.

The power inverter discrete command is provided by the control structure that is further developed below.

#### 3.2.1. Plant Modeling: PMSM and Power Inverter

**Figure 7:**
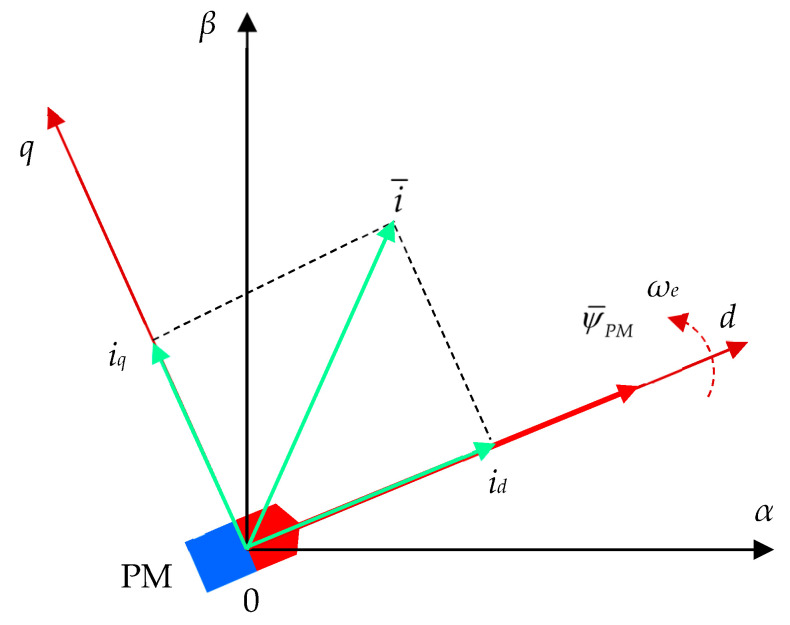
Space vector diagram of a PMSM with rotor system frame orientation.

Naturally, the model of PMSM is developed in the three-phase coordinates (*a*, *b*, *c*). This model has a complex structure and is difficult to use in practical closed-loop control applications. A first approach to avoid this drawback is to use orthogonal fixed coordinates (*α*,*β*) that offer some improvements. At last, the orthogonal transformation in a moving system reference frame (*d*,*q*) provides the highest benefits of the model of PMSM for closed-loop applications. Indeed, the usual FOC technique applied to PMSM is done by aligning the space vector of PM flux ${\bar{\psi }}_{PM}$ with the north pole of the PMSM rotor (Figure 7).

In addition, the stator current components (*i_d_*, *i_q_*) of the space vector current $\bar{i}$ admit to being colinear and perpendicular to the PM flux direction. Both space vectors PM flux ${\bar{\psi }}_{PM}$ and current $\bar{i}$ rotate with the electrical speed ω_e_.

For this kind of application, the pair of flow and effort mentioned above in the previous section corresponds to torque and angular speed:

(34) $$(e,s)\mapsto (m,\omega_{m}).$$

The PMSM model obtained by the FOC principle consists of the currents and the motion equations [8]:

(35) $$\begin{array}{l} \frac{di_{d}(t)}{dt}=\frac{1}{L_{d}}(u_{d}(t)-R_{s}i_{d}(t)+L_{q}\omega_{e}(t)i_{q}(t))\\ \frac{di_{q}(t)}{dt}=\frac{1}{L_{q}}(u_{q}(t)-R_{s}i_{q}(t)-L_{d}\omega_{e}(t)i_{d}(t)-\omega_{e}(t)\psi_{PM}) \end{array},$$

(36) $$\frac{d\omega_{m}(t)}{dt}=\frac{1}{J}\left(m(t)-\frac{\mu_{f}}{z_{p}}\omega_{m}(t)-m_{l}(t)\right),$$

where (*u_d_*, *u_q_*), (*i_d_*, *i_q_*), and (*L_d_*, *L_q_*) are voltages, currents, and inductances, all expressed in (*d*, *q*) coordinates, (ω_e_, ω_m_) are the electrical and mechanical angular speed, respectively, *R_s_* is the stator resistance, *ψ_PM_* is the permanent magnet flux, *m*, and *m_ℓ_* are the electromagnetic and load torques, respectively, and the group of constants given by *z_p_*, *J*, and *μ_f_* that are poles pairs, total inertia of drive and friction factor, respectively.

The system (35) and (36) were completed by introducing the expressions of the electromagnetic torque, magnetic flux components, input and output power, respectively, as follows:

(37) $$m(t)=\frac{3}{2}z_{p}\left[\left(L_{d}-L_{q})i_{d}(t)i_{q}(t)+\psi_{PM}i_{q}(t\right)\right],$$

(38) $$\begin{array}{l} \psi_{d}(t)=L_{d}i_{d}(t)+\psi_{PM}\\ \psi_{q}(t)=L_{q}i_{q}(t) \end{array},$$

(39) p1(t)=Re{u¯i¯*}=Re(ud(t)+juq(t))(id(t)−jiq(t))p2(t)=m(t)⋅ωm(t),

where $\bar{u}$ and $\bar{i}$ are the phasors of voltage and current, respectively, ${\bar{i}}^{*}$ represents the complex-conjugate phasor of current, and Re{◦} stands for the real part of the applied quantity.

**Figure 8:**
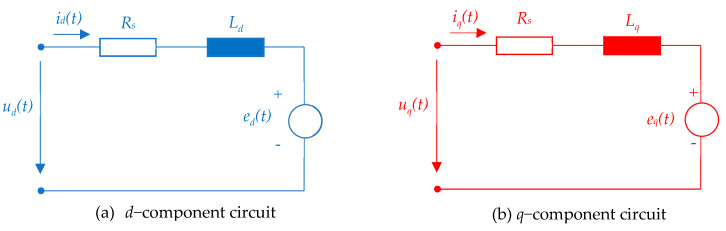
The *dq* equivalent circuit scheme of PMSM.

The equivalent circuit scheme of the PMSM based on the *dq* current model (35) is shown in Figure 8, where the total ElectroMotive Force (EMF) force components have been introduced:

(40) $$\begin{array}{l} e_{d}(t)=-L_{q}\omega_{e}(t)i_{q}(t)\\ e_{q}(t)=L_{d}\omega_{e}(t)i_{d}(t)+\omega_{e}(t)\psi_{PM} \end{array}.$$

The Equations (34)–(38) describe the so-called *dq* model of the PMSM drive system. Based on the *dq* model, the proper design of the control structure is performed. However, the physical operation of PMSM takes place in the natural three-phase system reference frame. To obtain the desired quantities, we used the Clarke and Park direct/indirect transformations, respectively, of the quantity λ = u, i, ψ, as follows:

(41) $$\begin{array}{c} \lambda_{\alpha \beta }=T_{\alpha \beta }\lambda_{abc}\\ \lambda_{abc}=T_{\alpha \beta }^{-1}\lambda_{\alpha \beta } \end{array},$$

(42) $$\begin{array}{c} \lambda_{dq}=T_{dq}\lambda_{\alpha \beta }\\ \lambda_{\alpha \beta }=T_{dq}^{-1}\lambda_{dq} \end{array},$$

where the vector-matrix quantities used are:

(43) $$\begin{array}{l} \lambda_{abc}={\left[\begin{array}{ccc} \lambda_{a} & \lambda_{b} & \lambda_{c} \end{array}\right]}^{T},\\ \lambda_{\alpha \beta }={\left[\begin{array}{cc} \lambda_{d} & \lambda_{q} \end{array}\right]}^{T},\\ \lambda_{dq}={\left[\begin{array}{cc} \lambda_{d} & \lambda_{q} \end{array}\right]}^{T}. \end{array},$$

and the involved matrix-transformations are given by:

(44) $$\begin{array}{c} T_{\alpha \beta }=\frac{2}{3}\left[\begin{array}{ccc} 1 & -\frac{1}{2} & -\frac{1}{2}\\ 0 & \frac{\sqrt{3}}{2} & -\frac{\sqrt{3}}{2} \end{array}\right];T_{\alpha \beta }^{-1}=\frac{2}{3}\left[\begin{array}{ccc} 1 & 0 & 1\\ 0 & -\frac{1}{2} & \frac{\sqrt{3}}{2} \end{array}\right];\\ T_{dq}=\frac{2}{3}\left[\begin{array}{ccc} \cos\alpha_{m} & \cos\left(\alpha_{m}-\frac{2\pi }{3}\right) & \cos\left(\alpha_{m}+\frac{2\pi }{3}\right)\\ \sin\alpha_{m} & \sin\left(\alpha_{m}-\frac{2\pi }{3}\right) & \sin\left(\alpha_{m}+\frac{2\pi }{3}\right) \end{array}\right];T_{dq}^{-1}=\frac{2}{3}\left[\begin{array}{ccc} \cos\alpha_{m} & \sin\alpha_{m} & 1\\ \cos\left(\alpha_{m}-\frac{2\pi }{3}\right) & \sin\left(\alpha_{m}-\frac{2\pi }{3}\right) & \frac{\sqrt{3}}{2} \end{array}\right]. \end{array}$$

The power inverter command will be supplied by the finite set values **S**_abc,_ which contain the optimal logical values required to obtain inverter control.

Based on finite set values **S**_abc_, the model of the two-level power inverter provides the three-phase voltages:

(45) $$u_{abc}=\frac{1}{3}U_{DC}T_{I}S_{abc},$$

where the power inverter matrix model is:

(46) $$T_{I}=\left[\begin{array}{ccc} 2 & -1 & -1\\ -1 & 2 & -1\\ -1 & -1 & 2 \end{array}\right].$$

#### 3.2.2. MPC with Finite Set

The DDC method is developed by using the main features of the MPC strategy that provide a command law corresponding to the voltage of the inverter computed by a switching function set. In this strategy, the main variables such as current, voltage, and electromagnetic torque are discretized at every instant $T_{s}.$ by the Euler forward method, which gives to a generic variable *z*(*t*) the next first-time derivative linear approximation:

(47) $${\left.\frac{dz(t)}{dt}\right|}_{z=z(k+1)}\approx \frac{z(k+1)-z(k)}{T_{s}},$$

Taking into account the Euler forward approximation method, the first-time derivative of current (35) becomes:

(48) $$\begin{array}{l} i_{d}(k+1)=\left(1-\frac{T_{s}R_{s}}{L_{d}}\right)i_{d}(k)+\frac{L_{q}}{L_{d}}T_{s}\omega_{e}(k)i_{q}(k)+\frac{T_{s}}{L_{d}}u_{d}(k)\\ i_{q}(k+1)=\left(1-\frac{T_{s}R_{s}}{L_{q}}\right)i_{q}(k)-\frac{L_{d}}{L_{q}}T_{s}\omega_{e}(k)i_{d}(k)-\frac{T_{s}}{L_{q}}\omega_{e}(k)\psi_{PM}+\frac{T_{s}}{L_{q}}u_{q}(k) \end{array},$$

Now, from the first-time derivative of current (37), the predictive electromagnetic torque is:

(49) $$m(k+1)=\frac{3}{2}z_{p}\left[\left(L_{d}-L_{q})i_{d}(k+1)i_{q}(k+1)+\psi_{PM}i_{q}(k+1\right)\right],$$

The one-step-ahead MPC law is an optimal problem solution defined as follows:

(50) $$\begin{array}{c} g^{*}=\underset{S_{abc}}{\min g_{i}}\\ s.t.\left\{\begin{array}{l} g_{i}=\left\{\begin{array}{l} \left|m^{ref}-m(k+1)\right|+i_{d}(k+1),\;\;\;\;\;\;\;\;\;\;\omega_{m}\leq \omega_{m}^{rated}\\ \left|m^{ref}-m(k+1)\right|+\left|emf^{rated}-(\psi_{PM}+L_{d}i_{d}(k+1))\right|,\;\;\omega_{m}>\omega_{m}^{rated} \end{array}\right.\\ i=1÷8;\;k=1÷N \end{array}\right.\; \end{array}.$$

where *emf*^rated^ is the rated back motion EMF computed by:

(51) $$emf=k_{e}\omega_{m}.$$

with the back EMF constant:

(52) $$k_{e}=z_{p}\psi_{PM},$$

the index *i* corresponds to the combination of switching state **S**_abc_ (see Table 1 below).

**Figure 9:**
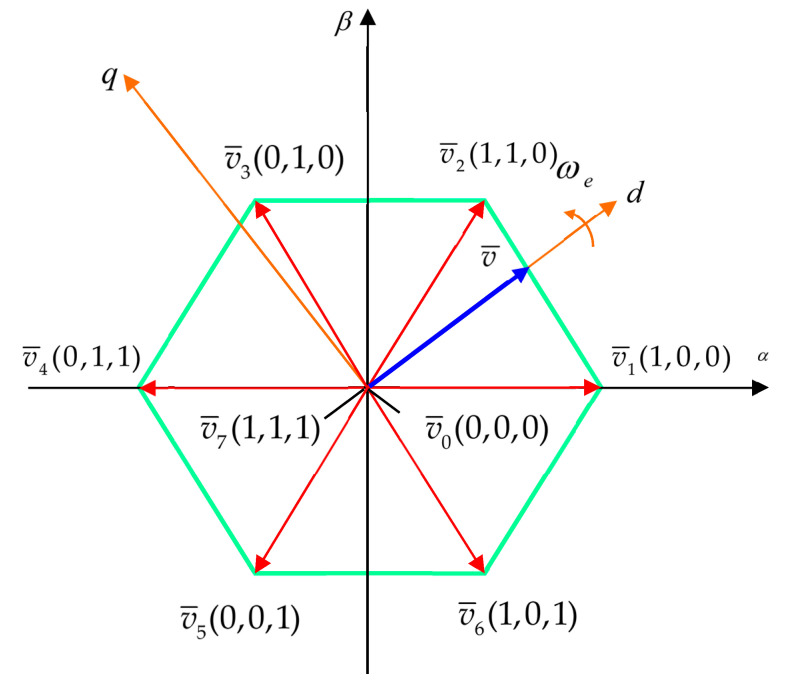
The voltage space vector according to their corresponding switching states.

A better illustration of the voltage space vector of the power inverter and its hexagonal bounds is shown in Figure 9.

In the *dq* system reference frame, the components of the space vector $\bar{v}$ for the eight possible states are obtained in a vector-matrix representation:

(53) $$\begin{array}{l} v_{d}=\left[\begin{array}{cccccccc} 0 & 2 & 1 & -1 & 2 & -1 & 1 & 0 \end{array}\right]\frac{U_{DC}}{3}\\ v_{q}=\left[\begin{array}{cccccccc} 0 & 0 & 1 & -1 & 0 & -1 & -1 & 0 \end{array}\right]\frac{\sqrt{3}}{3}U_{DC} \end{array}.$$

**Figure 10:**
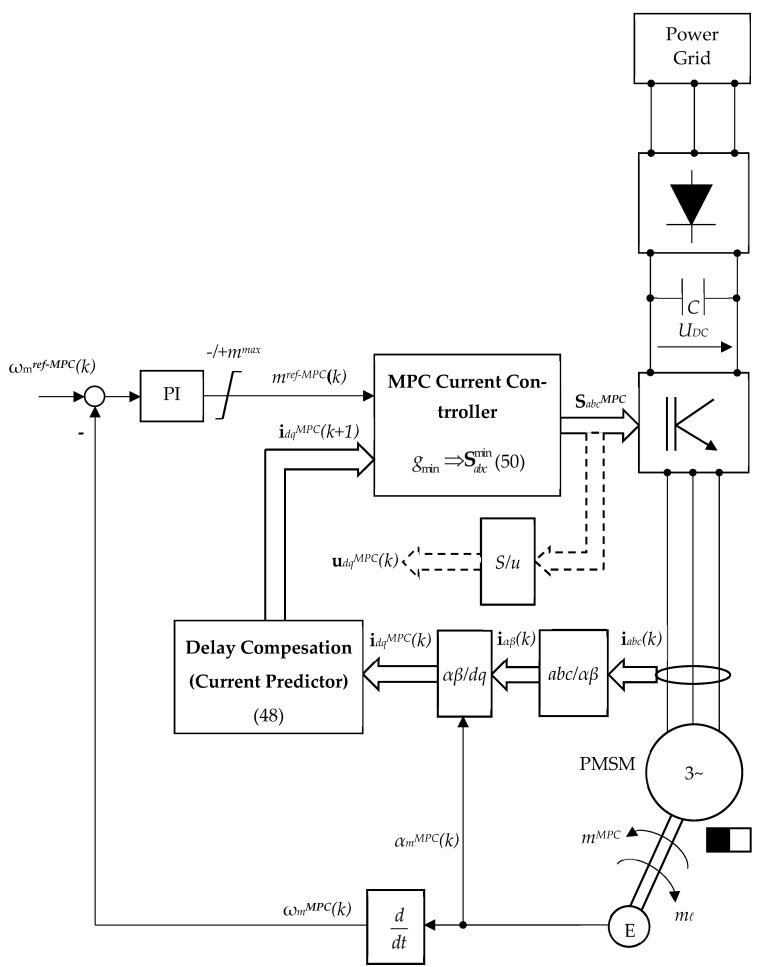
The MPC control PMSM drive.

Furthermore, Figure 10 shows the cascade control structure with a PI speed control in an outer loop and an MPC multivariable inner current control loop for the electronic power converter that supplies the PMSM. The PI speed control is designed via the classical pole placement method [45].

The electrical energy provided by the power grid is of fixed values of voltage and frequency. By means of a dedicated rectifier, a DC voltage is obtained. The DC-bus voltage U_DC_ is filtered by a passive Low Pass Filter (LPF) consisting of a high-value capacitor C, obtaining a constant value of the DC-bus voltage U_DC_ = cst.

The PI controller is used for tracking the mechanical speed *ω_m_^MPC^* its reference *ω_m_^ref−MPC^*. For the MPC formulation of the inner loop, the three-phase current (*a*, *b*, *c*) is measured, and then by the Park/Clarke transformations, the *dq* MPC current **i***_dq_^MPC^*(*k*) = [*i_d_^MPC^*(*k*) *i_q_^MPC^*(*k*)]^T^ are successively obtained. Next, based on (48) the current prediction **i***_dq_^MPC^*(*k +* 1) = [*i_d_^MPC^*(*k +* 1) *i_q_^MPC^*(*k +* 1)]^T^ is obtained. Now, the MPC problem defined according to (50) will yield optimal switching combinations *S_abc_^MPC^.* For future computations, the voltages **u***_dq_^MPC^*(*k*) = [*u_d_^MPC^*(*k*) *u_q_^MPC^*(*k*)]^T^ are additionally calculated based on a switch-voltage transformation *S/u*.

It can be mentioned that the mechanical speed *ω_m_^MPC^* is not directly measured. Firstly, the position of the rotor *α_m_^MPC^*(*k*) is measured by an encoder (E), and then the actual mechanical speed *ω_m_^MPC^*(*k*) is obtained by derivation.

#### 3.2.3. DDC Law of PMSM

The main idea of the DDC strategy is to learn from a well-known MBC method whose performances have already been proven. In this respect, the DDC method of a PMSM drive is developed by selecting a DB containing the data provided by the MPC law. Following the methodology developed for DDC in the sense of the maximum energy conversion process objective, for PMSM, the output is given by speed and torque $y(k)\equiv \left[\begin{array}{cc} \omega_{m}^{MPC}(k) & m^{MPC}(k) \end{array}\right]$, while the control by *dq* voltage is $u(k)\equiv u_{dq}^{MPC}(k)$. In this context, the training sequence becomes:

(54) $$d^{MPC}(k)={\left[\begin{array}{ccc} u_{dq}^{MPC}(k) & \omega_{m}^{MPC}(k) & m^{MPC}(k) \end{array}\right]}^{T},$$

Also, for the PMSM drive, the training matrix of the RBF-NN consists of the speed and *dq* voltage of the reduced model (high-efficiency points extraction):

(55) $$\begin{array}{l} M_{F}=[\begin{array}{cc} \overset{⏝}{y} & u \end{array}{]}^{T},\\ \overset{⏝}{y}=[\begin{array}{cc} {\overset{⏝}{\omega }}_{m}^{MPC}(k) & {\overset{⏝}{m}}^{MPC}(k) \end{array}{]}^{T},\\ u=u_{dq}^{MPC}(k), \end{array}$$

**Figure 11:**
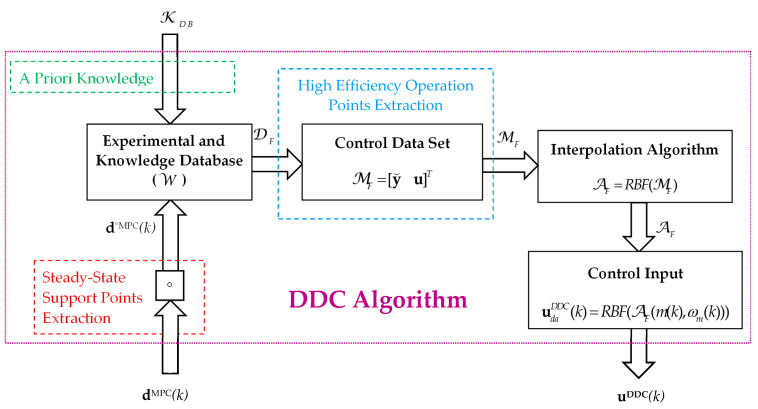
Training phase of the DDC controller of PMSM.

The training phase of the DDC controller is depicted in Figure 11.

As mentioned above, the entire methodology is based on learning from the experience of the high-value efficiency steady-state data provided by the MPC strategy.

The set of apriori information $K_{DB}$ includes rated data of PMSM, the power inverter load and other components, environment, and design requirements of EDSs.

**Figure 12:**
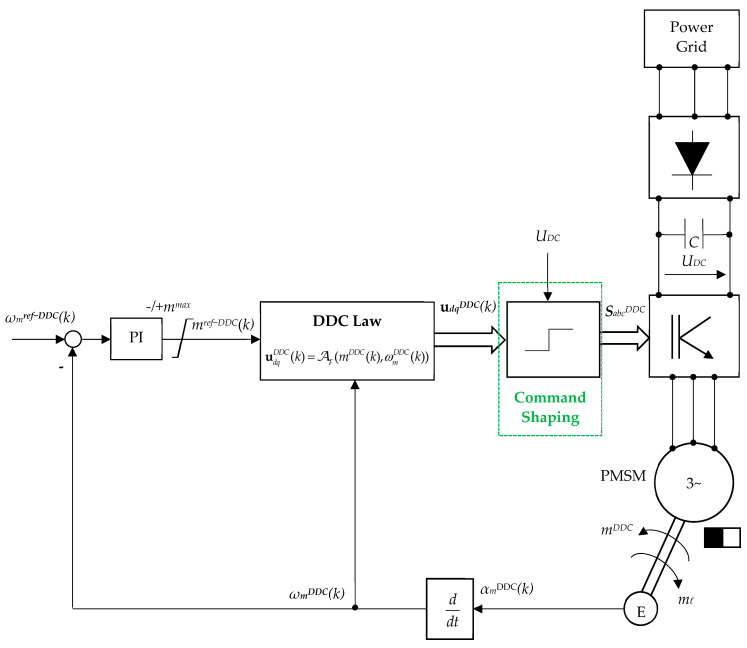
The DDC control structure of a PMSM drive.

Once the training is completed, the DDC control structure may be performed, as seen in Figure 12. The output of the DDC controller is given by the *dq* voltage $u_{dq}^{DDC}(k)$. The optimal set of the switching function combinations $S_{abc}^{DDC}$ is then obtained by a command shaping block.

**Figure 13:**
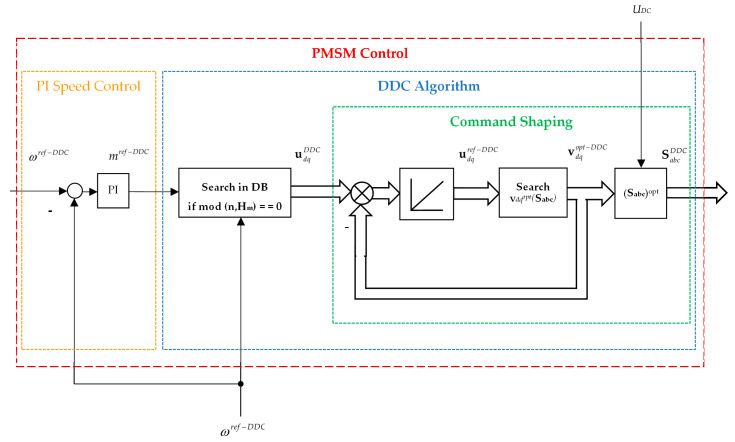
A schematic representation of the DDC control of the PMSM drive system.

To obtain a succinct description, Figure 13 depicts a schematic representation of the DDC control structure. The main controllers, PI and DDC, are highlighted. The DDC algorithm is represented in a sequential structure, where the command shaping has an internal computing loop. The DDC algorithm has a complex structure. The first stage is searching on the DB for the corresponding command $u_{dq}^{ref-DDC}$. Once found, an internal computing programming loop is designed. Then, the command $u_{dq}^{ref-DDC}(k)$ and the feedback $v_{dq}^{opt-DDC}(k)$ (that is also an optimal value) are compared, resulting in an error that is subsequently integrated into obtaining the voltage control $v_{dq}^{c-DDC}(k)$ that acts as input in the searching block. Finally, the switching functions developed by the DDC method $S_{abc}^{DDC}$ that will feed the gate of the inverter are obtained.

More details will be presented later in the pseudocode format of the DDC method.

#### 3.2.4. Design of the DDC Database

The input data in DB is initially done from the MPC operation, selected from the high-efficiency values. After that, the data are processed according to various criteria. Hence, the acronym MPC will be substituted, making the distinction between MPC and DDC whenever necessary.

The operation of a PMSM drive depends on the required mechanical speed domain limits. Generally, three situations are possible, showing how torque and outer power vary with the mechanical speed:

(56) $$\omega_{m}=\left\{\begin{array}{ll} I.\;\;\;\omega_{m}\in (0,\omega_{m,b}) & \Rightarrow \omega_{m}↗\Rightarrow \left.\left\{\begin{array}{l} m=cst.\\ P_{2}↗ \end{array}\right.\right|\mathrm{Constant}\;\mathrm{torque}\\ \mathrm{II}.\;\;\omega_{m}\in (\omega_{m,b},\omega_{m,\max}) & \Rightarrow \omega_{m}↗\Rightarrow \left.\left\{\begin{array}{l} m↘\\ P_{2}=cst. \end{array}\right.\right|\mathrm{Constant}\;\mathrm{power}\\ \mathrm{III}.\;\omega_{m}>\omega_{m,\max} & \Rightarrow \omega_{m}↗\Rightarrow \left.\left\{\begin{array}{l} m↘\\ P_{2}↘ \end{array}\right.\right|\mathrm{Critical}\;\mathrm{operation} \end{array}\right.,$$

where *ω*_*m*,*b*_ is the based mechanical speed that corresponds to the rated one (*ω*_*m*,*b*_ = *ω*_*mN*_), and *ω*_*m*,*max*_ is the maximum mechanical speed.

**Figure 14:**
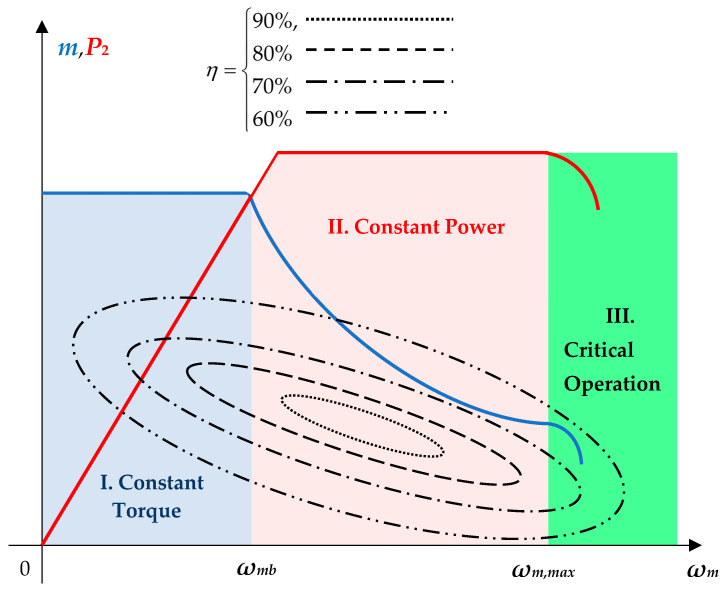
The torque-speed characteristics under iso-efficiency curves of a PMSM drive.

Figure 14 shows the torque-speed characteristics of a PMSM drive system. Up to the base speed *ω_m_* < *ω*_*m*,*b*_, the operation of PMSM is performed at constant torque in region I. In region II, where the power becomes constant, the field-weakening technique is used. Finally, the critical operation performed for *ω_m_* < *ω*_*m*,*max*_ must be avoided (region III) for safety reasons.

Let **Ω**, **M**, **P**, and **H** be the sets of sets of torque, power, and their corresponding efficiency:

(57) $$\Omega =\{\omega_{mj}|j=\bar{1,N_{\omega }}\},$$

(58) $$M=\{m_{j}|j=\bar{1,N_{m}}\},$$

(59) $$P=\{P_{2j}|j=\bar{1,N_{p}}\},$$

(60) $$H=\{\eta_{j}|j=\bar{1,N_{h}}\}.$$

where *N_ω/m/p/h_* is the number of points of each set.

As may be easily seen in Figure 14, some of the different pairs of speed-torque (*ω_mj_*, *m_j_*) or speed-power (*ω_mj_*, *P*_2*j*_) correspond to the same value of efficiency. That means that the solution is not unique.

Considering that all the sets (57)–(60) have the same number of points $N_{\omega }=N_{p}=N_{h}=N$, the next sets obtained by Cartesian product are introduced:

(61) $$\Lambda_{m}=\{\omega_{mj}\times m_{j}|j=\bar{1,N}\},$$

(62) $$\Lambda_{p}=\{\omega_{mj}\times P_{2j}|j=\bar{1,N}\}.$$

Now, the following multi-valued maps can be defined:

(63) $$f_{m}:\Lambda_{m}\to H,$$

(64) $$f_{p}:\Lambda_{p}\to H,$$

whose points are contained in the sets:

(65) $$\Gamma_{m}=\{(\Lambda_{m}(j),\eta_{j})|j=\bar{1,N}\},\;\;\Gamma_{m}\subseteq \Lambda_{m}\times H,$$

(66) $$\Gamma_{p}=\{(\Lambda_{p}(j),\eta_{j})|j=\bar{1,N}\},\;\;\Gamma_{p}\subseteq \Lambda_{p}\times H.$$

To avoid the multi-valued efficiency representation, we select the high-efficiency points that correspond to each point, defining the functions:

(67) $$f_{m}^{*}:\Lambda_{m}\to H^{\max},$$

(68) $$f_{p}^{*}:\Lambda_{p}\to H^{\max},$$

where the maximum efficiency vector is:

(69) $$H^{\max}={\left[\begin{array}{ccccc} \eta_{1}^{\max} & \eta_{2}^{\max} & \eta_{3}^{\max} & \cdots & \eta_{N}^{\max} \end{array}\right]}^{T},$$

and the contained sets are given by:

(70) $$\Gamma_{m}^{*}=\{(\Lambda_{m}(j),\eta_{j}^{\max})|j=\bar{1,N}\},\;\;\Gamma_{m}^{*}\subseteq \Lambda_{m}\times H^{\max},$$

(71) $$\Gamma_{p}^{*}=\{(\Lambda_{p}(j),\eta_{j}^{\max})|j=\bar{1,N}\},\;\;\Gamma_{p}^{*}\subseteq \Lambda_{p}\times H^{\max}.$$

**Figure 15:**
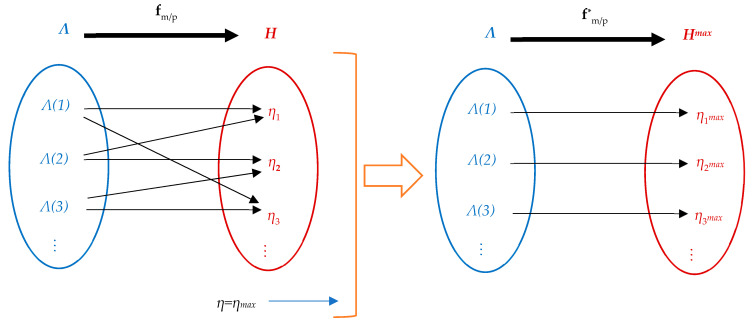
Transformation of the multi-valued efficiency map into a single-valued function.

An illustrative representation of the transformation of the multi-valued map of the efficiency vs. torque/power into a single-valued function of a PMSM drive characteristics is shown in Figure 15.

**Figure 16:**
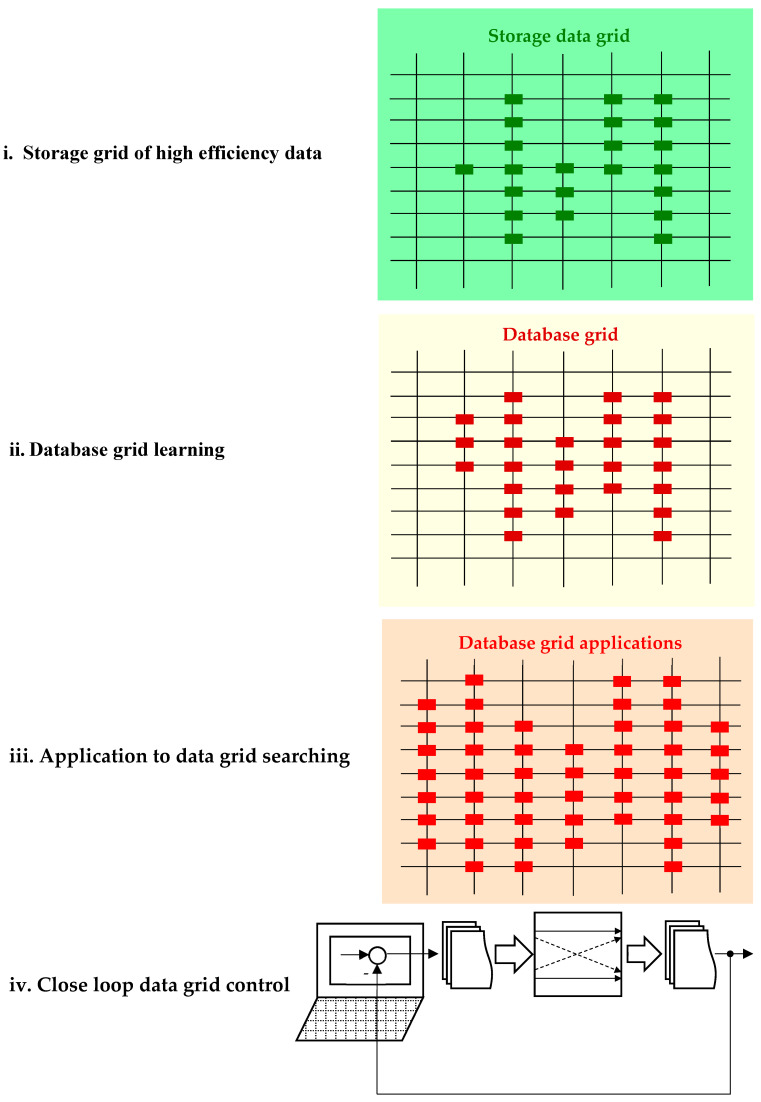
The database grid steps design of closed-loop applications.

An essential objective is the design of the DB. Generally, there are several steps that must be followed in order to obtain high computation accuracy, as suggested in Figure 16 by a classical approach.

Succinctly, the main followed steps can be described as follows:

- Storage grid of high-efficiency data. At this step, the main collected data set in the steady-state regime and knowledge, which corresponds to a high-efficiency value, is stored in $D_{F}=W\{K_{db};d^{{}^{\circ}}(k)\}$, with:
  (72) $$\begin{array}{l} d^{{}^{\circ}}(k)={\left[\begin{array}{cccc} {u^{{}^{\circ}}}_{dq}(k) & {i^{{}^{\circ}}}_{dq}(k) & m^{{}^{\circ}}(k) & {\omega^{{}^{\circ}}}_{m}(k) \end{array}\right]}^{T},\;\\ s.t.\;\eta (k)=\eta_{\max} \end{array}.$$
- Database grid learning. The DB set is extended by a learning process, adding new points contained in the set:
  (73) $$\begin{array}{l} d^{{}^{\circ}e}(k)⊇d^{{}^{\circ}}(k)\\ s.t.\;\eta (k)=\eta_{\max} \end{array}.$$
- Application to data grid searching. It is checked if the added new points correspond to the real operation for open-loop applications.
- Closed-loop data grid control. Finally, the designed database grid is introduced in the desired closed-loop application in Figure 12.

These steps can be changed/improved depending on the desired objectives. For example, exogenous quantities may have a high influence on the system, which is not in thermodynamic equilibrium.

The numerical computations of the quantities delivered by DB are affected by errors and noises. The DDC strategy is a discrete-interpolative searching method for the best solution within learning data bounds.

A moving window filter is used to increase data processing accuracy. The output of the filter is given by

(74) $$y^{ftr}[n]=\frac{1}{L_{w}}\sum_{k=0}^{L_{w}-1}u^{nftr}[n-k],$$

where *L_w_* is the filter length, *n* is the current sample, and the input samples (nonfiltered) and output samples (filtered), respectively, are:

(75) $$u^{nftr}={\left[\begin{array}{ccc} u_{dq}^{nftr}(k) & m^{nftr}(k) & \omega_{m}^{nftr}(k) \end{array}\right]}^{T},$$

(76) $$y^{ftr}={\left[\begin{array}{ccc} u_{dq}^{ftr}(k) & m^{ftr}(k) & \omega_{m}^{ftr}(k) \end{array}\right]}^{T},$$

where $u_{dq}^{nftr/ftr}(k)={\left[\begin{array}{cc} u_{d}^{nftr/ftr}(k) & u_{q}^{nftr/ftr}(k) \end{array}\right]}^{T}$.

**Figure 17:**
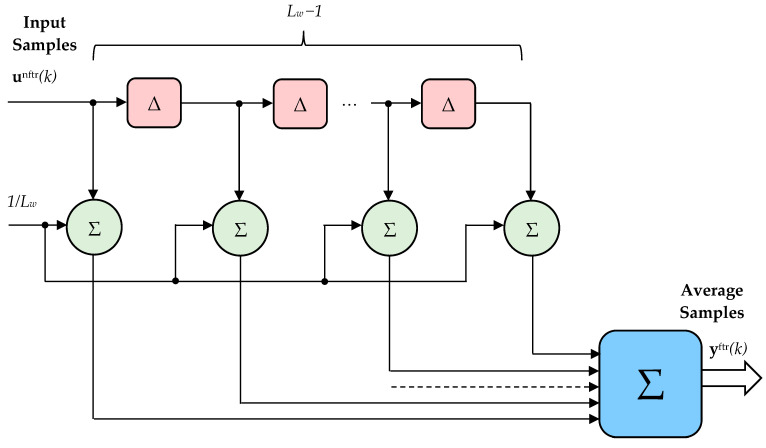
The moving window filter structure.

The moving window filter structure is shown in Figure 17. The input samples are introduced by using the unit-delay operator Δ. An average value of the moving samples is obtained for the output.

As can be observed from (75) and (76), the filtered quantities are voltage, torque, and mechanical speed. Based on this, further results in the filtering of both input and output powers of PMSM are obtained. Finally, the filtered powers are used to compute the efficiency, which does not contain high numerical noises and errors.

#### 3.2.5. DDC Matlab Implementation

The DDC of the PMSM is implemented in the Matlab simulation environment version R 2023b, taking into account the main programming features of this software package.

To obtain a high-quality analysis, the paper draws a comparison between the MPC method and the DDC strategy of the PMSM drive.

##### MPC Algorithm

The basic idea of the model predictive control for the PMSM fed by the simplest inverter with two voltage levels, having eight available state voltage switching vectors v*_i_* (Table 1), is the following: the torque and speed can be controlled by directly selecting a proper switching invertor’s voltage vector based on the machine parametric predictive model and an appropriate cost function *g.*

**Figure 18:**
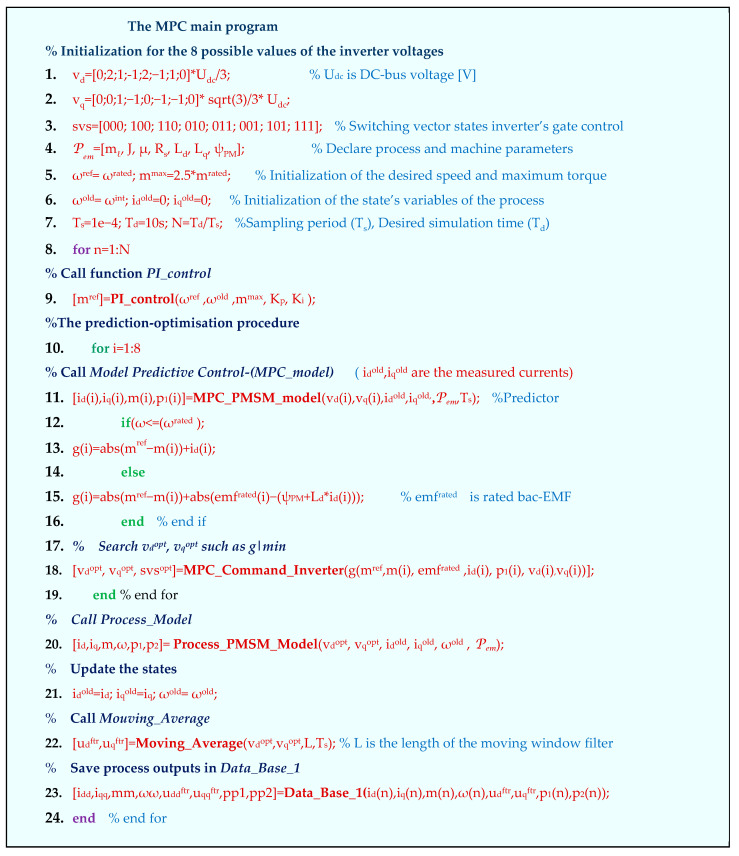
The pseudocode format of the MPC algorithm of a PMSM drive control.

The objective of algorithm, represented in pseudocode format in Figure 18, is to give the reader the necessary tools to understand the simulation results and to replicate the implementation of the exposed algorithms thinking in the Matlab environment.

The switching vector v^opt^, which was predicted to provide the most favorable process behavior, i.e., the one that minimizes the objective function *g*, is considered to be optimal and is generated by the inverter, applying the corresponding switch functions svs = [ S_a_, S_b,_ S_c_ ] directly.

The parameters of the motor and the driven process are included in the vector $P_{em}$ (electromagnetic parameters). After initializations of the finite states for the switching vector (lines 1, 2) and the rated values and state variables, the main iteration loop is opened (line 8).

The model requires the actual state variables $x=[\begin{array}{ccc} i_{d} & i_{q} & \omega_{m} \end{array}]$ to be recalled from the previous sampling period state $x^{old}=[\begin{array}{ccc} i_{d}^{old} & i_{q}^{old} & \omega_{m}^{old} \end{array}]$.

The electrical and mechanical parameters, respectively $P_{e}$ and $P_{m}$, of the plant are grouped into the electromechanical set of parameters $P_{em}$, as follows:

(77) $$P_{em}(t)==[\underset{P_{e}}{\underbrace{\begin{array}{cccc} R_{s} & L_{d} & L_{q} & \psi_{PM} \end{array}}}\underset{P_{m}}{\underbrace{\begin{array}{ccc} m_{l} & J & \mu_{f} \end{array}}}]=P_{e}\cup P_{m}.$$

The MPC algorithm starts by initializing the main data used in the computing loop.

A first programming syntax is developed for the implementation of the PI speed controller:

(78) $$\underset{\mathrm{Output}}{\underbrace{\left[m^{ref}\right]}}=PI\_control\underset{\mathrm{Input}}{\underbrace{\left(\omega^{ref},\omega^{old}\;m^{max},\;K_{p},\;K_{i}\right)}}$$

where $\omega^{ref/old}$ is the reference/actual mechanical speed $m^{max}$ is the maximal value of the electromagnetic torque, and *K_p/i_* are the parameters of the PI controller. **The PI_control** generates the required value of the torque reference (set point) *m^ref^.*

Relation (78) describes a typical programming syntax that contains the Input/Output sets of quantities. This type of programming syntax structure was applied in the subsequent sections of the paper.

The MPC strategy takes as input the voltages *v_d/q_*, the previously measured currents $i_{d/q}^{old}$, the electromechanical set of parameters $P_{em}(t)$ and the sampling time instant of MPC calculated around ten times higher than the simulation discretizing one:

(79) $$\left[i_{d}\left(i\right),i_{q}\left(i\right),m\left(i\right),p_{1}\left(i\right)\right]=MPC\_PMSM\_model(v_{d}\left(i\right),v_{q}\left(i\right),i_{d}^{old},i_{q}^{old},P_{em}(t),T_{s}).$$

Thinking of the Matlab environment implementation, the function that simulates the PMSM model has as the input the controls (*u_d_*, *u_q_*)—the components of the supply voltage, the old states, the load torque, and the electromagnetic parameters and as the outputs—the current components (*i_d_*, *i_q_*), the electromagnetic torque *m* and the angular velocity ω_m_:

(80) $$\underset{\mathrm{Output}}{\underbrace{\left[i_{d},i_{q},m,\omega \right]}}=Process\_PMSM\_model\underset{\mathrm{Input}}{\underbrace{\left[u_{d}^{old},u_{q}^{old},i_{d}^{old},i_{q}^{old},\omega^{old},m_{l},P_{em}(t),T_{s}\right]}}.$$

Finally, the programs were grouped and compacted in a software application of the proposed control structure.

The equations of the physical model (80) are in the continuous time domain. The variables of the model take values in the real domain ℝ. In fact, the PMSM is supplied by the electronic power inverter, which provides, in the simplest case, an eight-finite switching state vector:

(81) $$v_{dq}=\{v_{d}(i),v_{q}(i)\},\;\;i=1,\ldots ,8.$$

The command algorithm used by MPC control is implicit, which is different from the classical version. Within each sampling period *T_s_*, the command algorithm looks for the optimal components (*v_d_^opt^*, *v_q_^opt^*), which minimizes the *g* criterion function:

(82) $$[v_{d}^{opt},v_{q}^{opt},svs]=MPC\_Command\_Inverter\left[g\left(v_{d}(k),v_{q}(k),m,(k),m^{ref}(k),emf^{rated},i_{d}(k),p_{1}(k),T_{s}\right)\right].$$

This function provides the variables of the process at the current sampling period. The filter is implemented by the function Moving_Average, having as input the switching voltage vector components (*v_d_^opt^*, *v_q_^opt^*), the length of the window of the filter *L* and the sample time period *T_s_*, providing the output of the moving window that corresponds to the filtered inputs (*u_d_^ftr^*, *u_q_^ftr^*):

(83) $$\left[{u_{d}}^{ftr},{u_{q}}^{ftr}\right]=Mouving\_Average[{v_{d}}^{opt},{v_{q}}^{opt},L,T_{s}].$$

The numerical data calculated at each step *T_s_* are stored in the DB named Data_Base_1, which will continue to be used in the DDC Algorithm presented in the next section.

#### 3.2.6. DDC Algorithm

**Figure 19:**
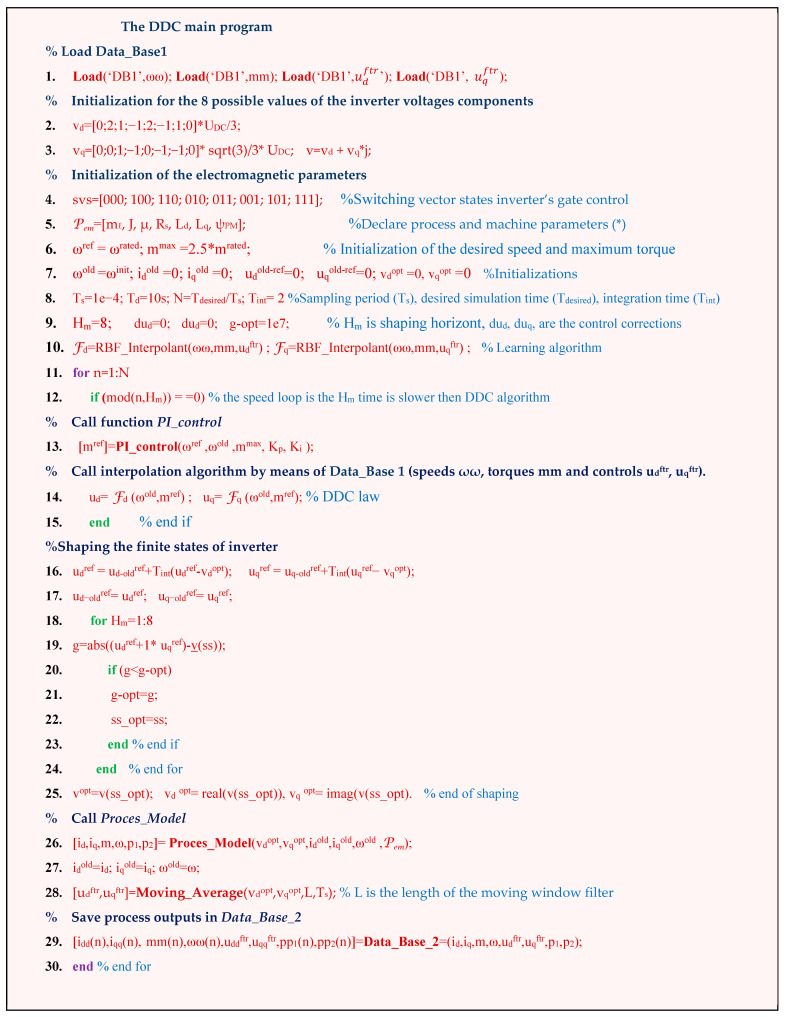
The DDC control algorithm of a PMSM drive control.

The DDC controls (*u_d_*, *u_q_*) are directly obtained from DB with $M_{F}$ (Figure 11) by the interpolation techniques RBF–NN treated in the second section. The control law was generated by employing a known set $M_{F}$ of operating points of the process (55) through an interpolation technique $A_{F}$ (25). The resulting algorithm of the DDC strategy in pseudocode format is shown in Figure 19 and schematically presented in Figure 13. The main common programming syntax from MPC and DDC has the same significance.

The data from database DB1 are loaded in the first line; they are created by means of the MPC algorithm (Figure 18), where the support points of the control surfaces are described by:

(84) $$\begin{array}{l} u(a)=F(mm(a),\omega \omega (a)),\\ u={\left[\begin{array}{cc} {u_{d}}^{ftr}(a) & {u_{q}}^{ftr}(a) \end{array}\right]}^{T},\\ F={\left[\begin{array}{cc} F_{d} & F_{q} \end{array}\right]}^{T},\\ \;a=1,\ldots ,N_{obs}. \end{array}$$

As mentioned before, the control system is designed in two stages. In the first stage, a set of motion trajectories are simulated by the MPC algorithm. The set of grided operation points of the process are stored in the database DB1.

Based on DB1, the DDC algorithm is simulated, yielding the results presented in the paper.

There is a particularity of the electric drive systems because the controls **u***_dq_*(k) must be shaped by the switching vector **v***_dq_* of the power inverter (81). The set $M_{F}$ of operating points of the process (55) is essential for the efficiency of the energy conversion system with a power electronic converter and electric machine. To this purpose, we selected out of a large database several sets of the optimal energy operation points defined by the speed, torque, voltage control of the power converter, and input and output powers.

The parameters P_em_ (line 5) are used for the offline simulation of the process. The online operation of the control algorithm is parameter-free. This is the main advantage of the DDC method.

In the main loop, the following functions are defined:

- The *PI_control*, which calculates the actual torque set point *m^ref^* using the measured past speed ω^old^ and the speed set point ω^ref^, and the tuning parameters K_p_ and K_i_;
- The matlab *RBF*_*Interpolant* function, which interpolates the control surfaces (24) by the scattered support points contained in DB 1;
- The function *Process_Model*, which calculates the actual process variables (i_d_, i_q_, m, ω,) and the powers *p*_1_, *p*_2_.

The control *u*(*a*) *= u_d_*(*a*) *+ ju_q_*(*a*) is the query of the actual points (*ω^old^*, *m^ref^*) for the surfaces $F_{d}$ and $F_{q}$. These controls are sampled from the continuous time domain data. The inverter is controlled by the finite state switching functions *svs*. The transformation of the controls *u_d_* and *u_q_* from a continuous time domain into a finite states domain of the inverter’s switching vectors *v_d_* and *v_q_* is a synthesis process.

The actual control *u*(*a*) is synthesized by a finite set of the switching vectors *v = v_d_ + jv_q_* as follows:

(85) $$\begin{array}{l} u(a)=\{{v_{k}}^{opt}(r),{v_{k}}^{opt}(r+1),\ldots {v_{k}}^{opt}(r+H_{m})\},\\ r=1,\ldots ,H_{m},\\ a=1,\ldots ,N_{obs}. \end{array}.$$

In (85), *H_m_* is the horizon of shaping, and each *v^opt^*(*k*) vector is determined by a searching procedure, minimizing the *g* criteria function. The dimension of the horizon *H_m_* was chosen by analogy with the classical MPC technique.

The components (*u_d_*, *u_q_*) take values within the set v*_dq_*. For this reason, these quantities must be shaped. According to the principle algorithm of the power inverter command, within each sampling period *T_s_*, the components (*v_d_^opt^*, *v_q_^opt^*) and the duration *d* (0 < *d* > *T_s_*) are calculated so that the machine currents resulting from switching supply voltage $v_{dq}^{opt}={\left[v_{d}^{opt},v_{q}^{opt}\right]}^{T}$ have to be as close as possible to the ideal sinusoidal form of the three-phase currents:

(86) $$Find:\;v_{dq}^{opt}\;\mathrm{until}\;\Rightarrow \;THDi_{abc}=\min,$$

The inverter command shaping function admits the syntax:

(87) $$[v_{d}^{opt},v_{q}^{opt}]=Inverter\_Command\_Shaping\left[u_{d}(k),u_{q}(k),T_{s}\right],$$

which is detailed and presented from 16 to 25 command lines.

## 4. Illustrative Case Study of the DDC of a PMSM Drive System

An industrial PMSM from a drive system supplied by a power inverter is used to illustrate the proposed DDC approach by comparison with the MPC control to check the optimal energy operation. The rated data of the PMSM drive are shown in Table 2.

The illustrative case study is performed using the Matlab-Simulink software version R 2023b in command-line implementation programs. For the simulation, the sampling period *T_s_* = 10^−4^ s was adopted. Also, the speed loop is designed by choosing a linear control law implemented by the PI controller designed by the pole placement method, resulting in the parameters *K_pω_* = 30 and *K_iω_* = 0.1. As apriori data $K_{DB}$, the PMSM parameters (Table 2), rated data for a conventional power inverter with DC link voltage *U_DC_* = 520 V are considered, the PMSM operating in a closed room.

The first objective is the design of the DB used for the DDC strategy based on the high-efficiency data collected from the MPC strategy running of PMSM. The second objective is to check the results of a comparative analysis obtained by MPC and DCC in rated conditions. To study the high impact of the DDC method, a third objective is to make a comparative analysis of the results obtained via MPC and DCC strategies in mismatch conditions. Finally, a comparative performance analysis of the previous results obtained via MPC and DDC strategies for rated and mismatched conditions is done in order to demonstrate that the developed DDC method in the paper is a relevant topic for future research.

### 4.1. Database Learning Design

The essential point in the DDC method is the DB design. First, there are the bootstrap DB obtained from process design theoretical data and parametric offline of the process.

From this extensive knowledge and DB, we extracted the control (working) DB. The control DB contains a few optimal efficiency steady-state operation points of the process (around 250) obtained in exogenous conditions. In our case, the exogenous conditions are the ambient temperature *T^0^* and the rated flux *ψ_PM_*. Taking into account the ambient conditions, the control becomes:

(88) $$u=A_{F}(\omega_{m},m,T^{o},\psi_{PM}).$$

As a result, the control DB must be extracted, and the RBF interpolation block will have four inputs and two output controls.

In this paper, we applied the direct searching corrections of the controls aiming at maximum energy efficiency. These corrections may be global or local.

A gridded experimental data point was obtained by simulating the MPC control algorithm, thus providing an admissible set of the operation points of the process selected in a steady-state regime for the highest efficiency values.

Global voltage corrections were adopted on the entire DB:

(89) $$\left\{\begin{array}{l} u_{d}^{DDC}=u_{d}^{MPC}k_{d}\\ u_{q}^{DDC}=u_{q}^{MPC}k_{q} \end{array}\right.,$$

where the factors *k_d_* = 1.04 and *k_q_* = 1 are selected to improve the DDC performances.

The local DB corrections are applied around a limited set of steady-state operational points. This kind of correction is applied mainly online in steady-state operation, in the sense of searching for the maximum energy efficiency in the actual exogenous conditions.

**Figure 20:**
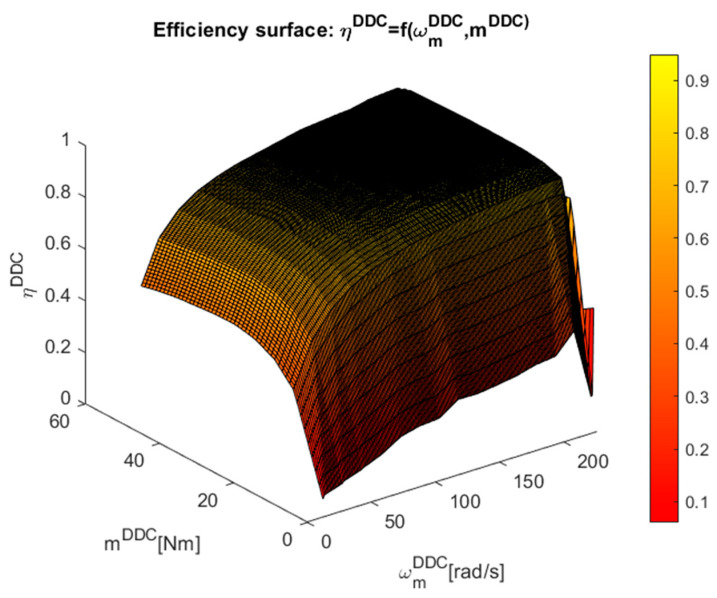
The efficiency surface vs. speed and torque.

The implicit discrete surfaces of the high-efficiency DDC control law $\eta^{DDC}=f(\omega_{m}^{DDC},m^{DDC})$, which include about 250 points of steady-state higher efficiency operation provided by the MPC strategy, were built in Figure 20 using this dataset.

**Figure 21:**
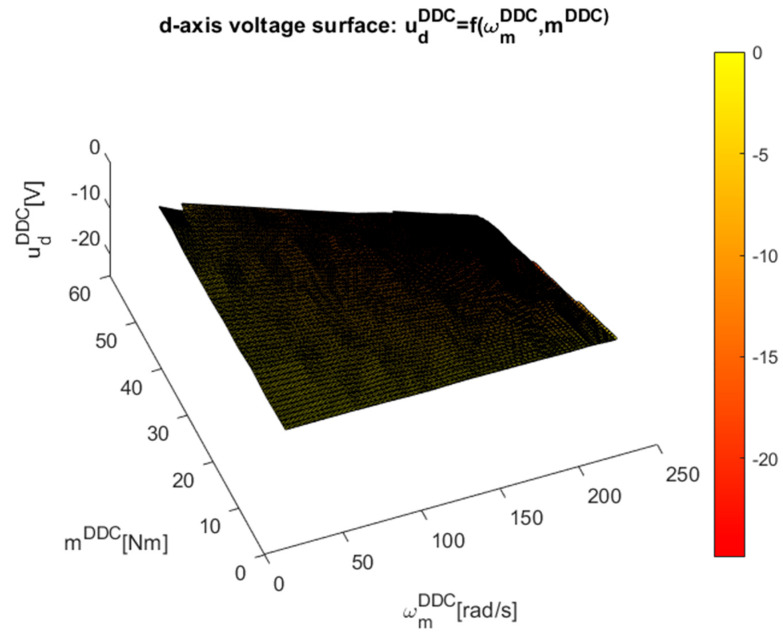
The *d*-axis voltage surface vs. speed and torque.

**Figure 22:**
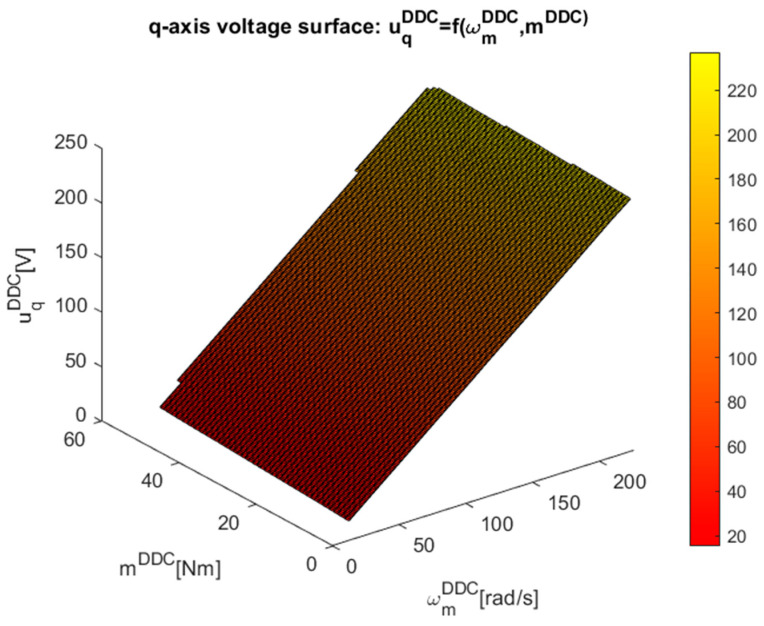
The *q*-axis voltage surface vs. speed and torque.

More attention must be paid to representing the control surfaces in Figure 21 and Figure 22 of the implicit control surfaces $u_{d/q}=f(\omega_{m}^{DDC},m^{DDC})$.

### 4.2. Study on Rated Data Conditions

This section provides a comparative analysis of the results obtained via MPC and DDC in rated conditions provided by the model parameters of PMSM in Table 2.

**Figure 23:**
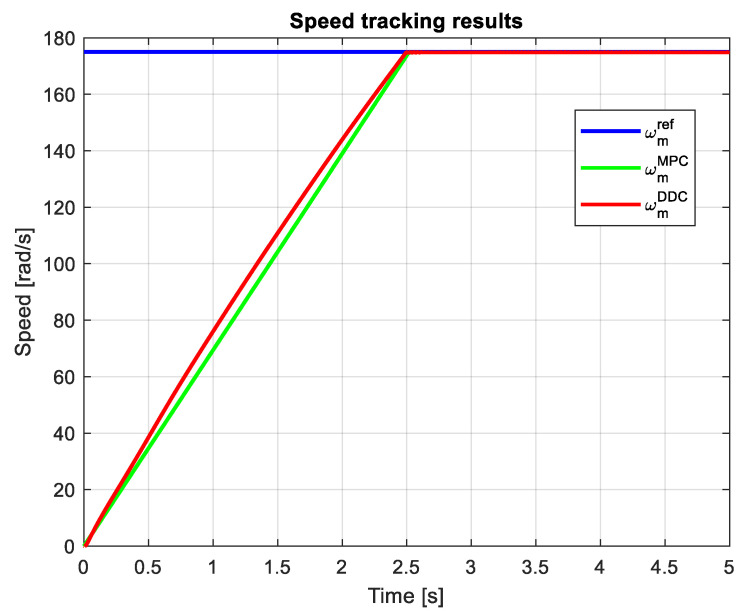
The speed-tracking results for MPC and DDC control strategies.

Adopting a speed step reference of $\omega_{m}^{ref}=175\;rad/s$, Figure 23 shows the speed tracking results obtained by MPC and DDC strategies. The speed provided by the DDC method $\omega_{m}^{DDC}$ and the MPC one $\omega_{m}^{MPC}$ yield rather similar responses, but the DDC has a sensible lower transient regime.

**Figure 24:**
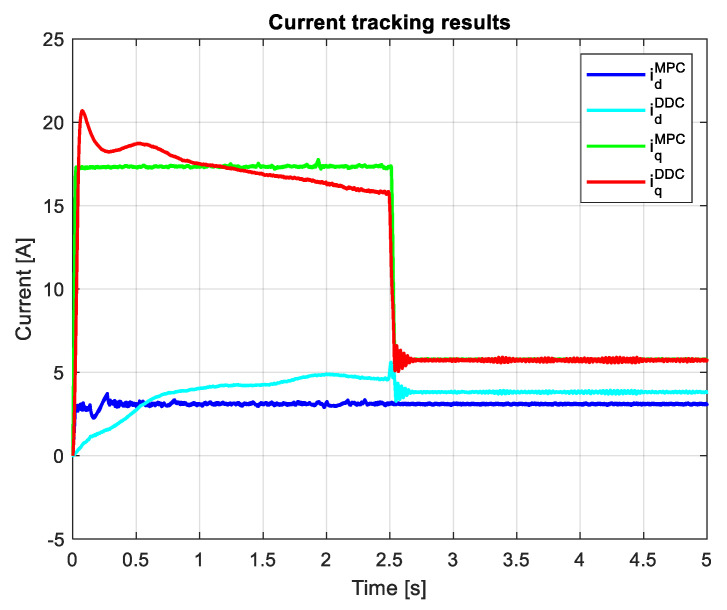
The current results obtained for MPC and DDC control strategies.

Figure 24 shows the current outcomes from the MPC and DDC strategies. In the case of the *d*-axis current component, the MPC law yields a smaller value than the corresponding one of the DDC law in absolute values $\left|i_{d}^{MPC}\right|<\left|i_{d}^{DDC}\right|$. After a different dynamic regime, the *q*-axis current component yields rather similar values in the steady state regime for both DDC and MPC controllers.

**Figure 25:**
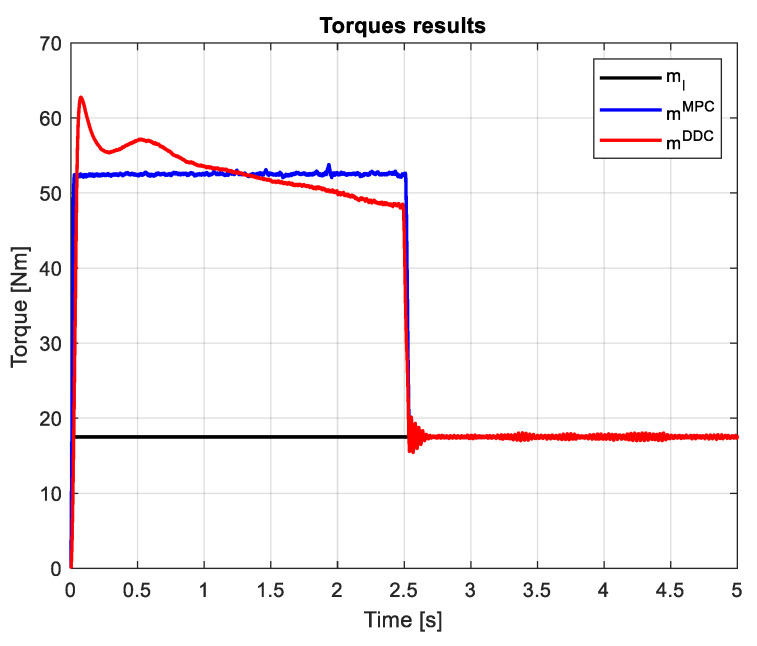
Load and electromagnetic torques for MPC and DDC control strategies.

A step load torque $m_{l}=17.5$ Nm is applied on the shaft of the PMSM drive. The load and electromagnetic torques provided by MPC/DDC laws are seen in Figure 25. Except for the startup process, in the dynamic regime, the electromagnetic torque obtained by DDC is lower than the one given by MPC $m^{DDC}<m^{MPC}$. However, the speed transient results in a higher dynamic torque regime for the DDC method.

**Figure 26:**
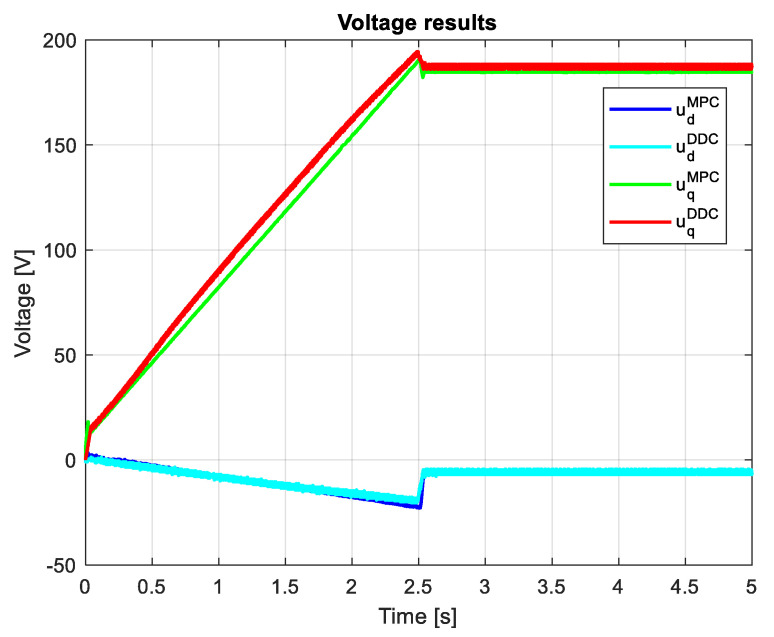
Voltage components for MPC and DDC control strategies.

Figure 26 indicates the voltages obtained by both MPC and DDC strategies $u_{d/q}^{MPC}$ and $u_{d/q}^{DDC}$. As a consequence of increasing the *q*-axis voltage component provided by DB, this difference is seen in the figure, having $u_{d}^{MPC}>u_{d}^{DDC}$. For the *d*-axis, the voltage component has similar values for MPC and DDC strategies.

**Figure 27:**
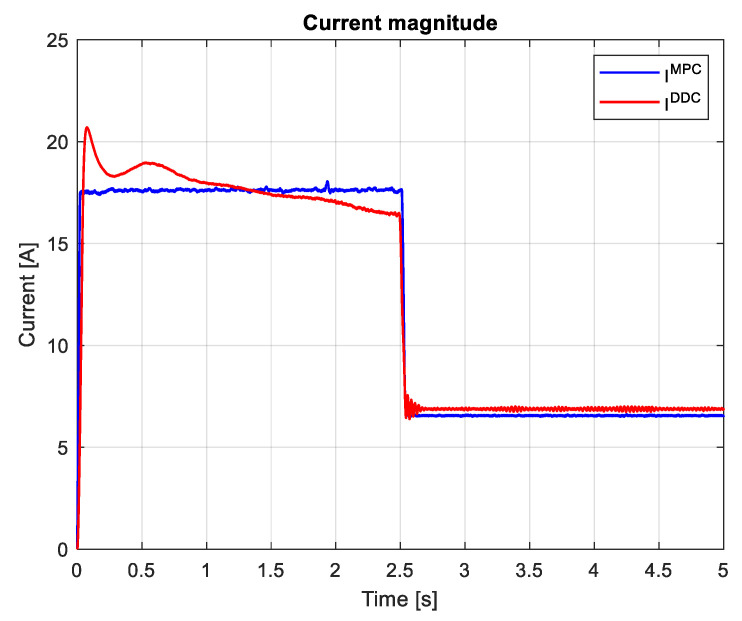
Current magnitude for MPC and DDC control strategies.

**Figure 28:**
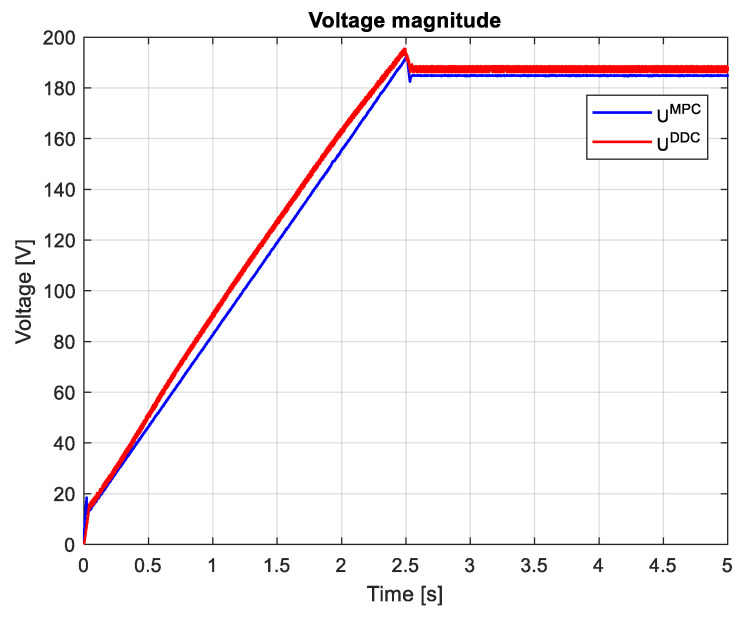
Voltage magnitude for MPC and DDC control strategies.

To have a clear assessment of the current tracking or voltage delivered by the control system of PMSM, in the following, we evaluated the current/voltage magnitude computed according to:

(90) $$I=\sqrt{i_{d}^{2}+i_{q}^{2}},$$

(91) $$U=\sqrt{u_{d}^{2}+u_{q}^{2}},$$

which are graphically shown in Figure 27 and Figure 28. It can be seen that, after the startup, during the transient regime, the DDC method provides a lower value than the corresponding one given by the MPC strategy $I^{DDC}<I^{MPC}$, and in the steady-state regime, it becomes higher $I^{DDC}>I^{MPC}$. As a consequence of (89), we always have $U^{DDC}>U^{MPC}$.

**Figure 29:**
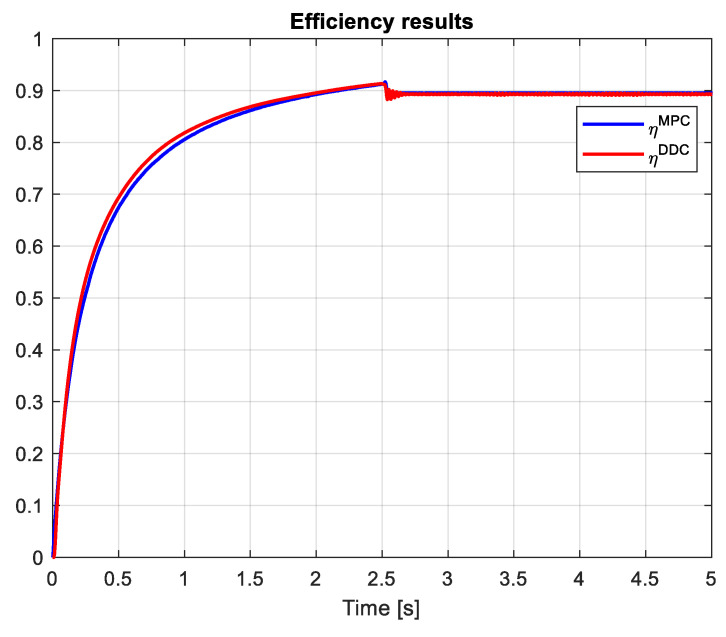
Efficiency of PMSM for MPC and DDC control strategies.

The efficiency of PMSM obtained by MPC and DDC control strategies is shown in Figure 29. As expected, the new control technology provides advanced benefits such as parameter-free and high-efficiency objectives. Thus, the dynamic efficiency compared to the classical one is higher $\eta^{DDC}>\eta^{MPC}$.

**Figure 30:**
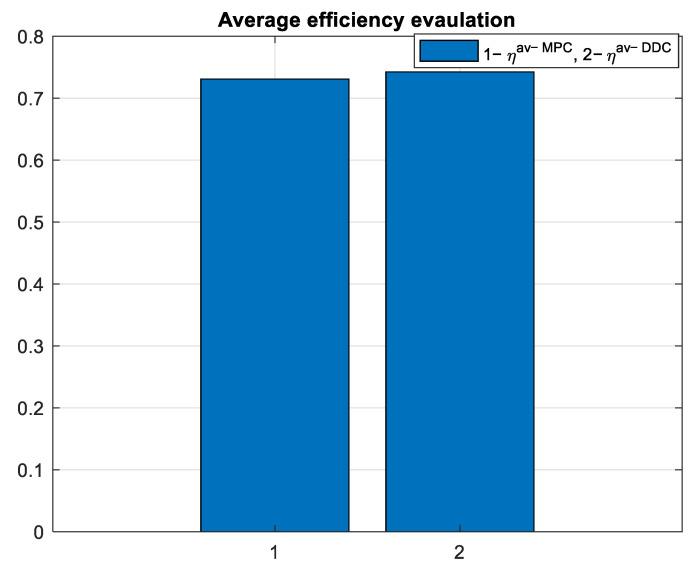
Average efficiency of PMSM for MPC and DDC control strategies.

To evaluate the efficiency of the PMSM on the entire simulation domain, we used the average efficiency defined according to:

(92) $$\eta^{av}=\frac{\sum_{a=1}^{N^{obs}}\eta (a)}{N^{obs}},$$

which is graphically represented in Figure 30.

**Figure 31:**
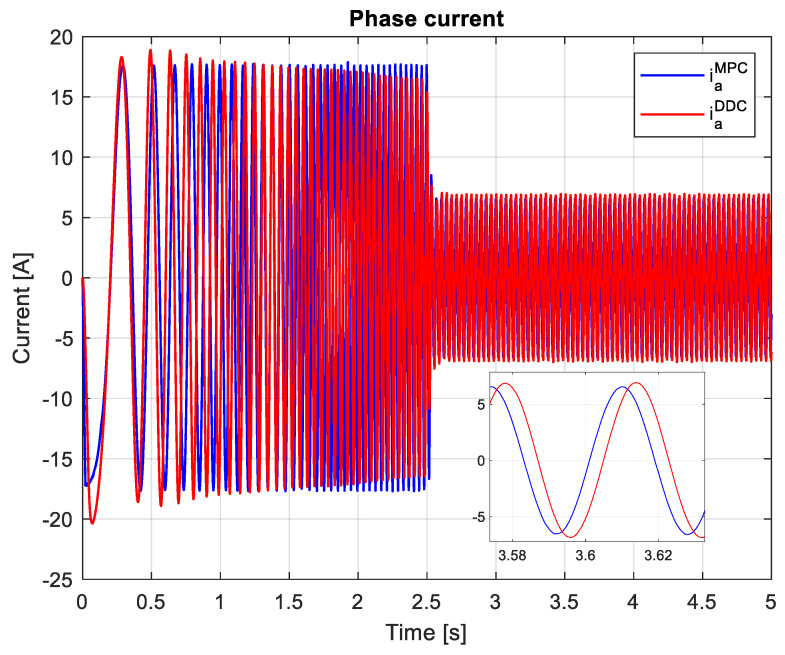
Phase current of PMSM for MPC and DDC control strategies.

Finally, it is important to assess the phase currents. Since the PMSM has a symmetric mechanical and electrical structure, the system of currents is also symmetrical, which is why only the current on the first phase $i_{a}^{MPC/DDC}$ will be shown, as in Figure 31. The peak of the phase current provided by the DDC method has a higher value than the one given by the MPC law.

The first conclusion is that the DDC strategy may operate without any mathematical model, being also parameter-free. The key to DDC strategy operation is related to database design, learning process, and available computational resources. A more important appreciation is that DDC improves both tracking references and efficiency. However, the DDC strategy remains stable in the imposed simulation scenario.

### 4.3. Study on Mismatch Conditions

Often, in practice, the *dq* PMSM model with constant lumped parameters is just an idealization that cannot always be implemented in real-life conditions.

In the following, the next variations of the parameters of the *dq* model of PMSM are considered due to demagnetization and temperature increasing, respectively, as follows:

(93) $$\left\{\begin{array}{l} \psi_{PM}\to 0.8\psi_{PM}\\ R_{s}\to 1.4R_{s} \end{array}\right.,$$

otherwise, all the conditions remain unchanged.

**Figure 32:**
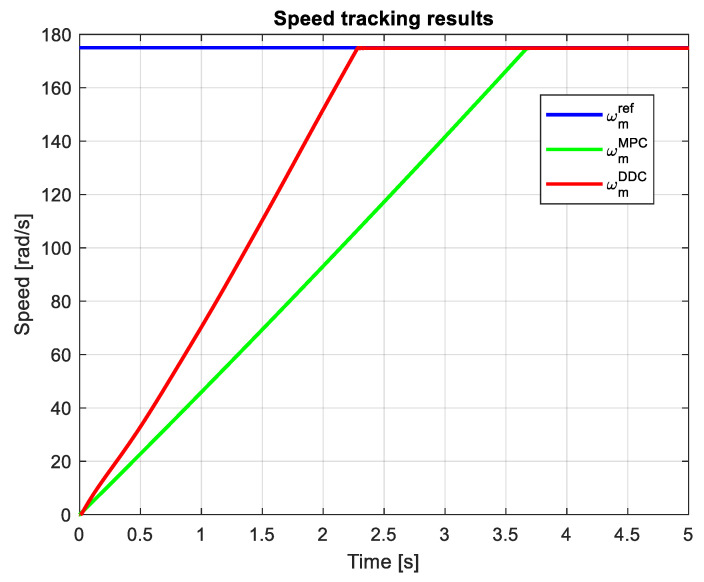
The tracking results of speed obtained via MPC and DDC control laws.

In this case, the speed tracking results obtained by MPC and DDC strategies are shown in Figure 32. Now, again, the speed obtained by the DDC strategy $\omega_{m}^{DDC}$ has a shorter dynamic regime than the one given by MPC law $\omega_{m}^{MPC}$. The speed response is even better than the one obtained in the rated scenario. For the MPC strategy, the results get worse with the parameters mismatch.

**Figure 33:**
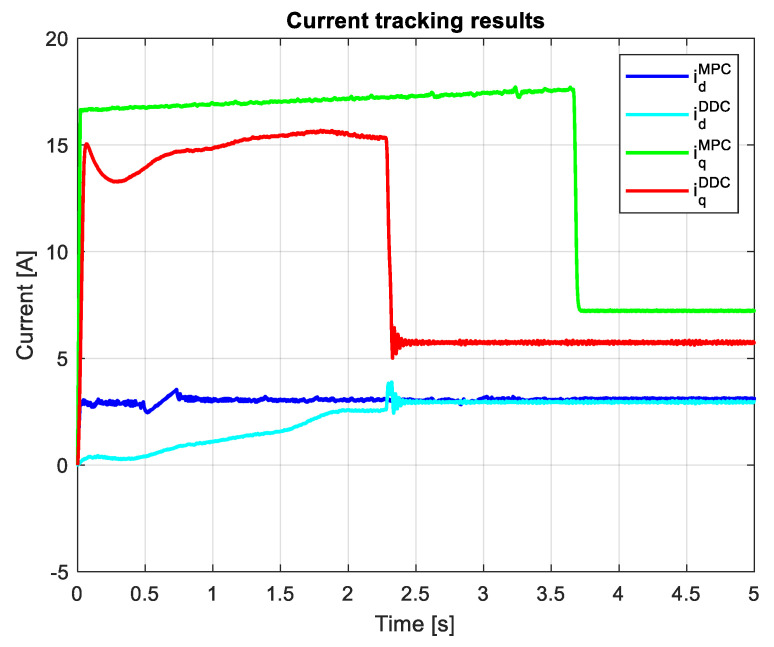
The *dq* current obtained for MPC and DDC control laws.

For current results given by MPC and DDC laws, both transient and steady-state regimes are different, as seen in Figure 33. The *d*-axis current component delivered by the MPC controller and the one of DDC law have different values in the dynamic regime and are close in a steady-state regime. On the *q*-axis, the current given by MPC and DDC has different values in both dynamic and steady-state regimes.

**Figure 34:**
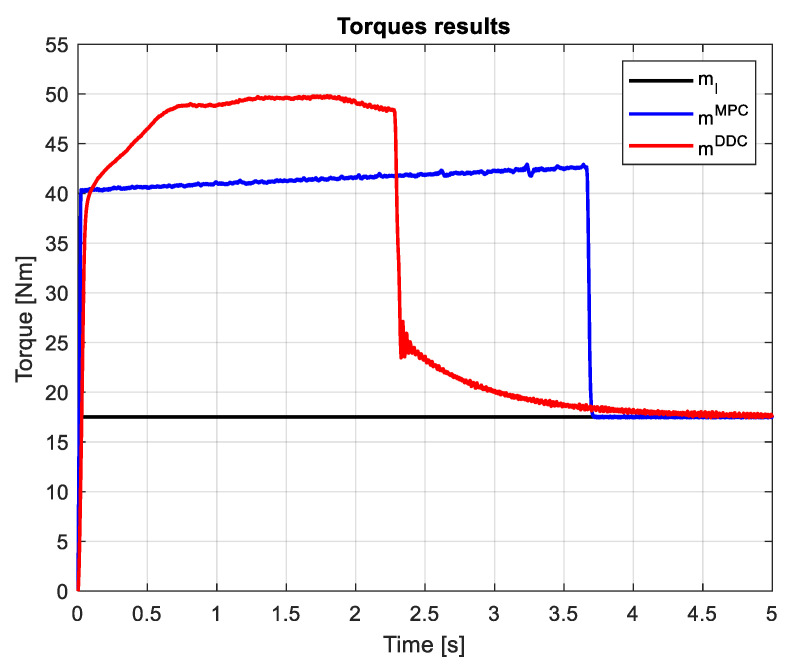
Load torque disturbance rejection for MPC and DDC control strategies.

Applying the same step load torque $m_{l}=17.5\;Nm$, the load and electromagnetic torques obtained by MPC/DDC strategies have different dynamics, as seen in Figure 34. The dynamic torque given by DDC has a higher value than the one provided by MPC $m^{DDC}>m^{MPC}$, but acts in a short transient. Thus, the evolution of the electromagnetic torques occurs with different dynamics until the main disturbance consisting of the load torque is rejected.

**Figure 35:**
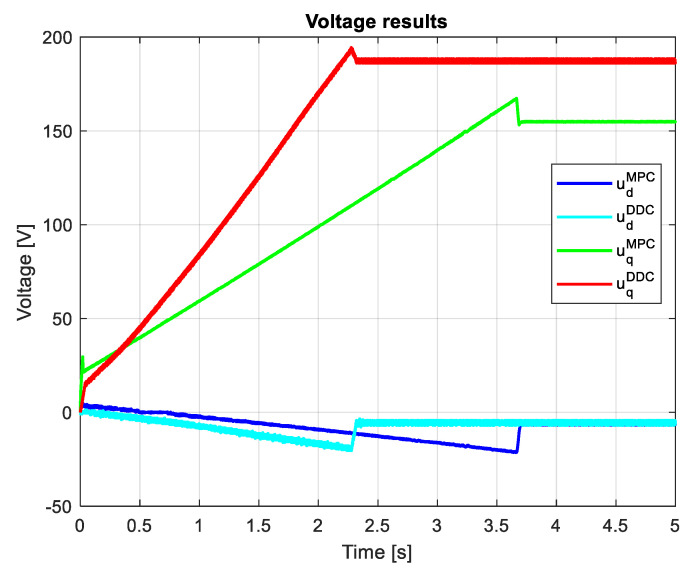
Voltage obtained by MPC and DDC control strategies.

The voltages provided by both MPC and DDC strategies $u_{d/q}^{MPC}$ are $u_{d/q}^{DDC}$ shown in Figure 35. On the *d*-axis, the voltage components are close in value, with small differences in the dynamic regime. Important differences are found on the *q*-axis, where $u_{d}^{DDC}>u_{q}^{MPC}$ has a significant value, mainly in steady-state regimes.

**Figure 36:**
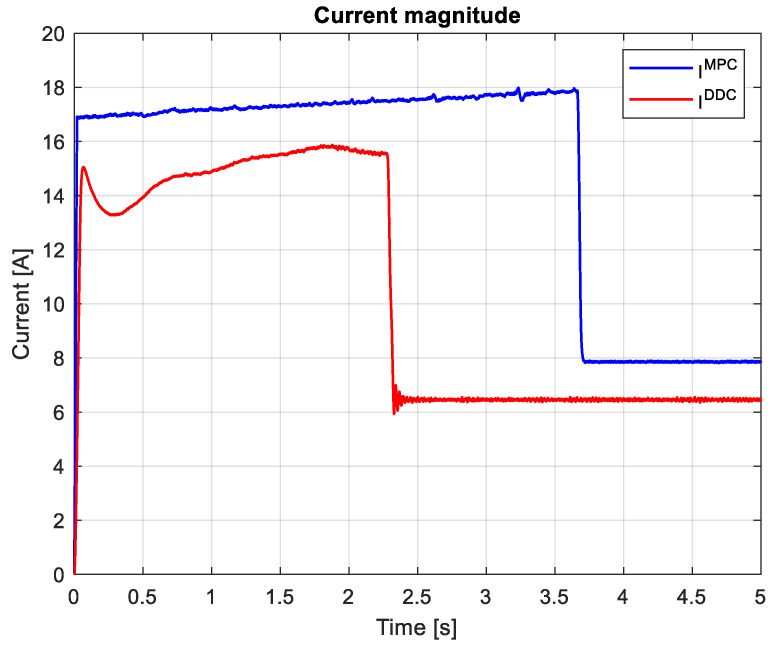
Current magnitude given by MPC and DDC control algorithms.

**Figure 37:**
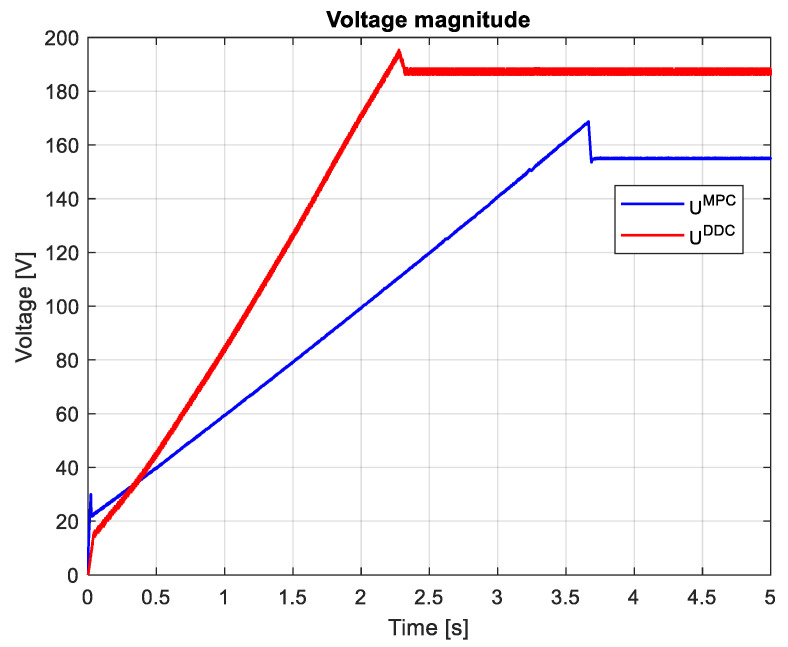
Voltage magnitude given by MPC and DDC control algorithms.

The magnitudes of current and voltage are shown in Figure 36 and Figure 37, respectively. The current magnitude provided by the MPC algorithm is always higher than the one obtained by the DDC law $I^{DDC}<I^{MPC}$. There is a significant increase in the voltage magnitude for the DDC algorithm, followed by the MPC controller. $U^{DDC}>U^{MPC}$.

**Figure 38:**
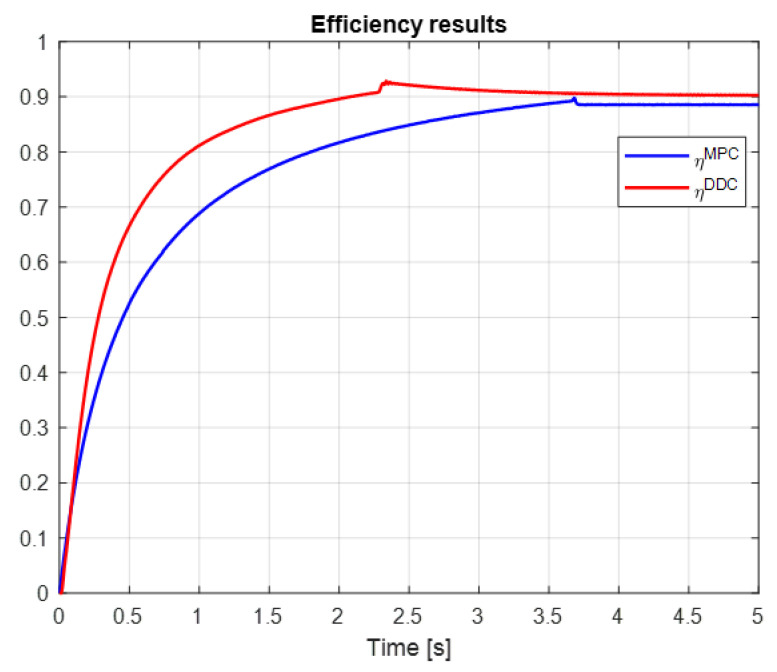
The efficiency of PMSM obtained by using MPC and DDC control algorithms.

The efficiency of PMSM obtained by the DDC law is substantially increased when comparing the MPC and DDC strategy $\eta^{DDC}>\eta^{MPC}$ in both dynamic and steady-state regimes, as illustrated in Figure 38. This is a consequence of the fact that the DDC method is designed by using the high-efficiency data collected from the MPC operation. This is a major objective in this robust scenario.

**Figure 39:**
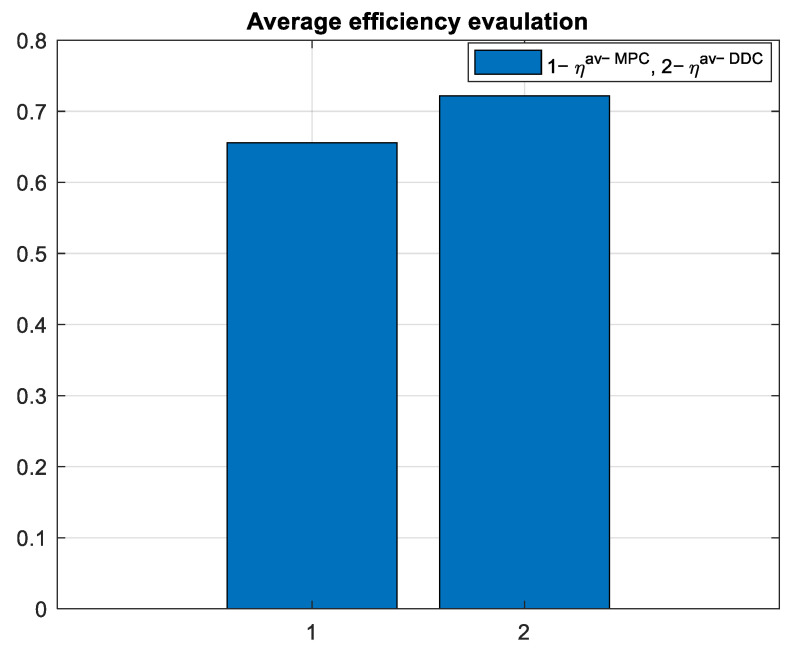
Average efficiency of PMSM provided by using MPC and DDC control algorithms.

As expected, the average efficiency for the entire simulation time is higher for the DDC method $\eta^{av-DDC}>\eta^{av-MPC}$, as shown in Figure 39.

**Figure 40:**
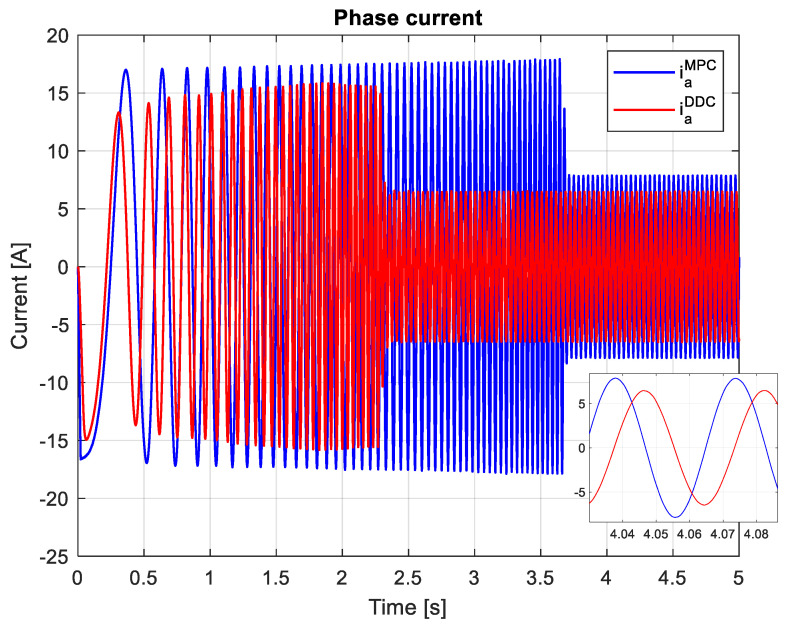
Phase current for MPC and DDC control algorithms.

In this scenario, the phase current obtained by the DDC strategy has a lower peak value than the one delivered by the MPC algorithm in both transient and steady-state regimes, as shown in Figure 40. However, a small peak value will generate reduced power losses, hence increasing the efficiency of PMSM. The DDC strategy is designed by using database learning that does not contain overcurrents, a feature that will maintain the phase current within a minimal range.

The research in this section demonstrates the outcomes of the DDC technique in the presence of PMSM parameter mismatch robustness situations. The primary goal of efficiency increase was proven to be reached. Nevertheless, the secondary goal is a reduction in the dynamic speed tracking regime. In contrast to the MPC approach, the DDC law needs higher voltage magnitude/components, which must account for the PMSM drive’s voltage limit levels. Additionally, by using the DDC approach, the phase current’s peak value is reduced, which results in decreased power losses.

### 4.4. Comparative Analysis of MPC and DDC Results

The proposed DDC method proves to have a stable operation in the rated and mismatch cases, and it also offers several benefits. For a relevant comparison of the cases discussed previously, the following criteria are used:

- Settling time of the mechanical speed—*t^st^*;
- Average efficiency on the entire simulation time—*η^av^*;
- Peak value of the *a* phase current in steady-state regime—*I_a_^pk^*;
- THD of the *a* phase current computed in the steady-state regime for ten harmonics, including the fundamental of the frequency 55.66 Hz, for a common time domain pf MPC and DDC strategies, 4–5 s time.

The comparative results based on these criteria of the MPC and DDC strategies are summarized in Table 3.

It is obvious that in both tests when compared to the MPC law, the DDC strategy leads to decreasing the settling time of the mechanical speed $t^{st-DDC}<t^{st-MPC}$. In rated conditions, a small difference occurs, but in the mismatch test, the DDC method leads to a setting time that is 61% lower than the one obtained by the MPC strategy, which is a high advantage.

For rated conditions, the increase in the average efficiency is small for the DDC strategy $\eta^{av-MPC}>\eta^{av-DDC}$, being around 1.2% from the one that resulted in rated conditions with the MPC controller. A high increase in the DDC average efficiency $\eta^{av-DDC}>\eta^{av-MPC}$, about 11%, results from the mismatch test, which is a major success in achieving the desired objective. In addition, the peak value of the phase current for rated conditions is lower for DDC, $I_{a}^{pk-DDC}<I_{a}^{pk-MPC}$, and higher in the mismatch case $I_{a}^{pk-DDC}>I_{a}^{pk-MPC}$. Consequently, through interpolation and digital signal processing, the phase current’s THD achieved by DDC law remains low in both the rated and mismatch cases under investigation.

We can conclude that the DDC strategy works better in the high-efficiency objective in both rated and robust scenarios than the classical MPC law. Nevertheless, the settling of speed responses also improved in both tests.

Finally, it should be noted that the results of the performances summarized in Table 3 are open to improvement since the results depend on the learning process and the available software and hardware resources.

## 5. Conclusions

The many faces of macroscopic energy conversion systems can be characterized by a set of generalized variables, such as effort and flow. This approach allows for the development of a physics-based mathematical equation model aimed at increasing the efficiency of energy conversion.

Despite the high performance of current devices and actuators for energy transformation, there is still plenty of room for efficiency improvement, especially at operating points with low effort and high flow or high effort and low flow. A transition from MBC to MFC is necessary to ensure a non-sensitive control approach. This study aims to construct the surfaces of the DDC law in terms of effort and flow.

Starting from a large knowledge DB, we created a control DB containing reasonable optimal efficiency steady-state operation points for the process. An RBF interpolation system was trained to extract from these surfaces the controls required for any operating point. This indicates that control efficiency is largely dependent on the consistency of the collected data.

AC drives are a field that emphasizes the fast processes developed in industry and residential applications. The performance of this DDC method was evaluated based on a PMSM drive system simulated by means of MPC methodology. The DB was built in a MATLAB simulation environment using a specific algorithm for MPC control of the PMSM drive and the DDC algorithm. The comparison criteria were the efficiency of energy process conversion (the ratio of output power to input power), the quality of control for speed and effort, and the THD of current. Due to variations in PMSM winding resistance and the degree of magnetic circuit depreciation over time, the DDC efficiency control is superior to MPC control because the MPC is dependent on model parameters that vary over time. Another benefit of the DDC strategy developed in this paper is that it does not require flux-weakening control together with a maximum criterion on the operation above the rated speed range, as was the case in classical approaches.

Many research papers in the field have demonstrated that the direct DDC strategy is better at reducing bias, while the indirect MPC strategy provides better variance. The results shown in Figure 27 and Figure 36 confirm that the MPC control provides better variance, while DDC offers better efficiency and adaptability to process parameter modifications.

Our DDC technique, being parameter-free, is more general than classical process model control methods such as MPC, as it does not depend on the nature of the process (electromagnetic, thermal, or mechanical). For AC drives, the DDC technique applies to all types of motors (asynchronous, synchronous, and others). The DDC method, when appropriately designed and managed, enables a high level of energy transformation quality characterized by fast dynamic response, high energy efficiency, and low parametric sensitivity.

The multiple aspects discussed and successfully addressed in this paper demonstrate that the novel technology developed herein remains an open problem that has the potential to yield realistic improvements in future promising approaches.

## Figures and Tables

**Table 1:** The voltage space vector according to their corresponding switching function.

Index *i*	S_abc_	Voltage Space Vector
1	(0,0,0)	${\bar{v}}_{1}=0$
2	(1,0,0)	${\bar{v}}_{2}=2/3U_{DC}$
3	(1,1,0)	${\bar{v}}_{3}=1/3U_{DC}+j\sqrt{3}U_{DC}$
4	(0,1,0)	${\bar{v}}_{4}=-1/3U_{DC}+j\sqrt{3}U_{DC}$
5	(0,1,1)	${\bar{v}}_{5}=-2/3U_{DC}$
6	(0,0,1)	${\bar{v}}_{6}=-1/3U_{DC}-j\sqrt{3}U_{DC}$
7	(0,1,0)	${\bar{v}}_{7}=1/3U_{DC}+j\sqrt{3}U_{DC}$
8	(1,0,1)	${\bar{v}}_{8}=0$

**Table 2:** PMSM-rated specifications data.

Symbol	Description	Values
*P_N_* [W]	Rated power	5500
*U_N_* [V]	Rated voltage	325
*m_N_* [Nm]	Rated torque	35
*I_N_* [A]	Rated current	10.6
*ω_mN_* [rad/sec]	Rated speed	157
*R_s_* [Ω]	Rotor resistance	0.65
*L_d_* [H]	Direct axis inductance	0.0082
*L_q_* [H]	Quadrature axis inductance	0.0082
*J* [kg∙m^2^]	Total inertia of the PMSM drive	0.5
*z_p_*	Stator pole pairs	2

**Table 3:** The comparative evaluation of MPC and DDC strategies.

Test	Control Strategy	*t^st^* [s]	*η^av^*	*I_a_^pk^* [A]	*THD_a_* [*%*]
Rated conditions	MPC	2.52	0.730	6.55	0.06
	DDC	2.50	0.742	6.96	0.17
Mismach conditions	MPC	3.68	0.612	7.86	0.08
	DDC	2.28	0.722	6.44	0.13

## Data Availability

Data are contained within the article.

## Abbreviations

AC	Alternative Current
AIMs	Artificial Intelligence Methods
DB	DataBase
DDC	Data Driven Control
DC	Direct-current
EDSs	Electric Drive Systems
EMF	ElectroMotive Force
HVAC	Hydro, Ventilation, Air Conditioning
MBC	Model Based Control
MFC	Model Free Control
MPC	Model Predictive Control
NN	Neural Network
PI	Proportional-Integral controller
LPF	Lower Pass Filter
PMSM	Permanent Magnet Synchronous Machine
RBF	Radial Basis Function
RL	Reinforcement Learning
THD	Total Harmonic Distorsion
QP	Quadratic Programming
WB	White-Box
**Nomenclature**	
(*a*, *b*, *c*)	Three phase coordinates
$A_{F}$	Set of the weights of the RBF-NN
(*α*, *β*)	Fixed coordinates
*α*_m_	Electric position of the rotor
***d^MBC^/^MPC^***	Set of processing data obtained by MBC/MPC
***d^oMBC^/^MPC^***	Steady-state set of processing data obtained by MBC/MPC
***d^oe^***	Set of data obtained by grid learning
***C_c_***, ***C_d_***	Continous/discrete cost
$D_{F}$	DB of knowledge and data of the process
Δ	Unit-delay
*e*	Effort
*e_d/q_*	*d/q* electrical EMF components
$E_{f,RMS},E_{n,RMS}$	RMS value of fundamental/ Harmonics components of the effort e
***E*** *_N_*	Set of envirovment data
*E*_1_, *E*_2_	Input/output energy
*emf*	Motion ElectroMotive Force (EMF)
*ε*	Mean square error
*η*, *η^av^*	Efficiency/Average efficiency
*ξ*	Discrete noise of the process state model
***F***	Operator of control law
***f***, ***g***	Nonlinear function of the state representation
*F_i_*	Explicit control law
*f_ε_*	Implicit represenation of the process model
*f_u_*	Useful force
*f*_1*/*2_	Components of surface grid representation
***f*** *_m/p_*	Multi-valued map of torque/output power
***f ^′^*** *_m/p_*	Single-valued map of torque/output power
*ψ_PM_*	Permanent magnet flux
*φ_i_*	Gaussian distribution
***Φ*** *^MBC/MPC^*	Training data set given by MBC/MPC
*g*	Cost function
*G_i_*	Implicit control law
*g*_1_, *g*_2_	Generalized variables of trajectories
***Γ_m/p_***	Set of speed and torque/output power and efficiency
***Γ ^′^_m/p_***	Set of speed and torque/output power and maximum efficiency
*h/**h***	Output of the space state model
*H_m_*	Horizon of command shaping searching
***H/H^max^***	Set of efficiency/maximum efficiency
***H*** *_N_*	Set of design data
*i*	Index that corresponds to the combination of switching
(*i_d_*, *i_q_*)	Direct and quadrature current components
$\bar{i}$	Current phasor
***I_a_^pk^***	Peak value of the a phase current
I	Input of the RBF-NN
*J*	Motal inertia
*k_d/q_*	Corection factor of voltage
*k_e_*	Back motion EMF constant
$K_{DB}$	Knowledge data
*l*	Index of the number of hidden neurons of the RBF-NN
(*L_d_*, *L_q_*)	Direct and quadrature inductances
*L_w_*	Filter length
*LPF*	Lower Pass Filter
***λ***	Generalized variables for transformation of PMSM model
***Λ_m/p_***	Set of the cartesian product of speed and torque/output power
*m*, *m_ℓ_*	Electromagnetic/load torque
***M***	Set of electromagnetic torque
$M_{F}$	Set of training data for RBF-NN
$K_{db}$	Set of the aprioric knowledge
*μ*	Center points of Gaussian function
*μ_f_*	Friction factor of the load
*N*	Number of trajectory points
*N_ω/m/p/h_*	Number of points of the set of speed,torque,power,efficiency, respectively
*N_obs_*	Number of observation points
*Q*	Flow rate
***θ***	Vector of parameters of the process state model
*P*	Pressure
**P**	Set of output power
*p*_1_, *p*_2_	Input/output power
$P_{e}$	Set of electrical parameters
$P_{m}$	Set of mechanical parameters
$P_{em}$	Set of electromechanical parameters
***R*** *_N_*	Set of rated data
*R_s_*	Stator resistance
*RMS*	Root mean square
Re{},Im{}	Real/imaginary part of a given qunatity
*s*	Flow
**S**_abc_	Set of the switching states
σ	Basis width vector of the Gaussian distribution
*t*	Time
*t*_0_, *t*_1_	Start/end time
*T_d_*	Desired final time
*T_s_*	Sampling time period
*T* ^0^	Temperature od stator winding of PMSM
$T_{\alpha \beta },T_{\alpha \beta }^{-1}$	Direct/Indirect matrix of the Clarke transformation
$T_{dq},T_{dq}^{-1}$	Direct/Indirect matrix of the Park transformation
$T_{i}$	Power inverter matrix
***u***	Control input
$\hat{u}$	Any other control then the optimal one
$\overset{⌣}{u}$	Control obtained via grid surface interpolation tehnique
**u**_abc_	Three-phase voltages
**u**_dq_	dq voltages
**u** ^ftr^	Filter input
**u** ^RBF^	Output of the RBF-NN
*u*	Voltage phasor
*U_DC_*	DC bus voltage
(*u_d_*, *u_q_*)	Direct and quadrature voltage components
(*u_d_^ftr/nftr^, u_q_^ftr/nftr^*)	Filtred/non-filtred commande of the power inverter
$\bar{u}$	Linear velocity
**v**_d/q_	Vectors of d/q components of power inverter
$v_{dq}^{c/opt-DDC}$	Command/optimal voltage of the DDC algorithm
*ω_e_*, *ω_m_*	Electrical/mechanical angular speed
*ω_m_*_,_ *_b_*	Based mechanical speed
*ω_m_*_,_ *_max_*	Maximal mechanical speed
*ω_mN_*	Rated mechanical speed
**x**	State of the state model
**y**	Output of the state model
$\overset{⌣}{y}$	Output of the interpolation method
**y** ^ftr^	Filter output
*z*	Generic variable used for the appling of the forward Euler forward method
*z_p_*	Stator magnetic poles pairs
*w*	Weights
W	Database operator of data processing
**Ω**	Mecahical speed set
